# Efficient synthesis of 3,6,13,16-tetrasubstituted-tetrabenzo[*a*,*d*,*j*,*m*]coronenes by selective C–H/C–O arylations of anthraquinone derivatives

**DOI:** 10.3762/bjoc.16.51

**Published:** 2020-03-31

**Authors:** Seiya Terai, Yuki Sato, Takuya Kochi, Fumitoshi Kakiuchi

**Affiliations:** 1 Department of Chemistry, Faculty of Science and Technology, Keio University, 3-14-1 Hiyoshi, Kohoku-ku, Yokohama, Kanagawa 223-8522, Japan

**Keywords:** C–H arylation, C–O arylation, oxidative cyclization, polycyclic aromatic hydrocarbons, ruthenium catalyst

## Abstract

An efficient synthesis of tetrabenzo[*a*,*d*,*j*,*m*]coronene derivatives having alkyl and alkoxy substituents at the 3, 6, 13, and 16-positions was achieved based on the ruthenium-catalyzed coupling reactions of anthraquinone derivatives with arylboronates via C–H and C–O bond cleavage. The reaction sequence involving the arylation, carbonyl methylenation, and oxidative cyclization effectively provided various tetrabenzo[*a*,*d*,*j*,*m*]coronenes in short steps from readily available starting materials. Tetrabenzo[*a*,*d*,*j*,*m*]coronenes possessing two different types of substituents were obtained selectively by sequential chemoselective C–O arylation and C–H arylation. The ^1^H NMR spectra of the tetrabenzo[*a*,*d*,*j*,*m*]coronene product indicated its self-assembling behavior in CDCl_3_.

## Introduction

Polycyclic aromatic hydrocarbons (PAHs) and their derivatives have attracted much attention from researchers due to their high potential as organic optoelectronic materials [1–3] and great efforts have been devoted to the development of their efficient synthetic methods [4]. One of the important steps in most of the PAH syntheses is the construction of carbon–carbon bonds between aryl groups to form biaryl frameworks. Traditionally, transition-metal-catalyzed cross-coupling reactions of aryl halides or pseudohalides with arylmetal reagents have been employed for the connection of two aryl units [5–9]. However, in search of more efficient synthetic routes, C–H arylation reactions have been increasingly utilized in recent years for the rapid expansion of the aromatic frameworks [10–15].

We have been working on the efficient syntheses of PAH derivatives based on the ruthenium-catalyzed C–H/C–O arylation reactions developed in our group [16–33]. In the presence of ruthenium carbonyl phosphine complexes, the reaction of aromatic ketones possessing C–H bonds or C–OR (R = alkyl) bonds with arylboronates provided C–H or C–O arylation products [21–33]. Anthraquinone was chosen as a convenient template for the PAH syntheses, because various anthraquinone derivatives possessing zero to four oxygen substituents at the *ortho*-positions are readily available and the regioselective arylation at the positions of either C–H or C–O bonds provided a variety of multiarylated anthraquinone derivatives [16–20]. Using this method, we have synthesized various π-extended aromatic compounds such as multiarylated acenes [16,18,20], dibenzo[*h*,*rst*]pentaphenes and dibenzo[*fg*,*qr*]pentacenes [19].

**Scheme 1:**
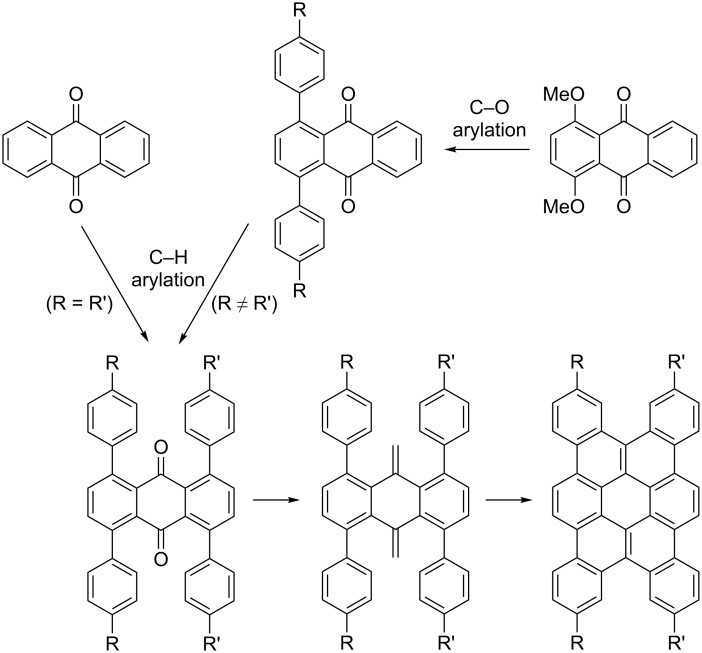
Projected synthetic routes for 3,6,13,16-tetrasubstituted tetrabenzo[*a*,*d*,*j*,*m*]coronenes.

In the course of our reaction development, it was found that an introduction of two different aryl groups at the *ortho*-positions can be achieved by chemoselective C–O arylation of aromatic ketones possessing both C–H and C–O bonds and subsequent C–H arylation. We envisioned that the application of this strategy to the anthraquinone template would provide its derivatives possessing two different types of aryl groups in a designed manner and lead to the preparation of tetrabenzo[*a*,*d*,*j*,*m*]coronenes having two different types of substituents (Scheme 1). While 1,4,5,8-tetraarylanthraquinones prepared by the ruthenium-catalyzed C–H arylation of anthraquinone with a *para*-substituted arylboronate may be converted to 3,6,13,16-tetrasubstituted tetrabenzo[*a*,*d*,*j*,*m*]coronenes via carbonyl methylenation and oxidative cyclization, the two-step sequential C–O and C–H arylation of 1,4-dimethoxyanthraquinone followed by subsequent methylenation/cyclization would provide tetrabenzo[*a*,*d*,*j*,*m*]coronenes having two different types of substituents.

**Scheme 2:**
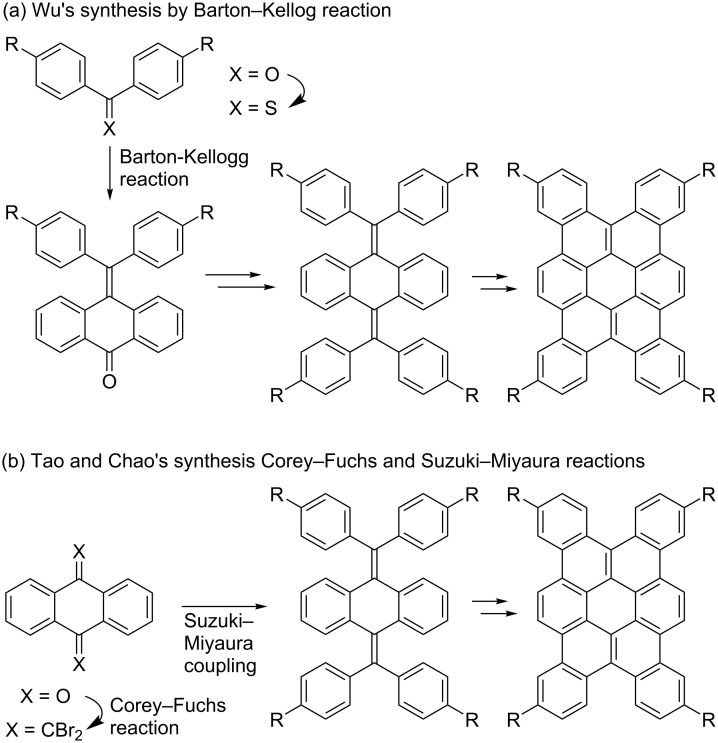
Reported syntheses of 2,7,12,17-tetrasubstituted tetrabenzo[*a*,*d*,*j*,*m*]coronenes.

Coronene derivatives such as hexabenzocoronenes and ovalenes have been extensively studied because of their optoelectronic and self-assembling properties [34–44]. Various synthetic methods have been developed for coronene derivatives in general [34–37], however, only a few strategies have been reported for the syntheses of tetrabenzo[*a*,*d*,*j*,*m*]coronenes [43–44]. Wu and co-workers reported the synthesis of 2,7,12,17-tetrasubstituted tetrabenzo[*a*,*d*,*j*,*m*]coronenes by using the Barton–Kellogg reaction, followed by dehydrogenative photocyclization, and FeCl_3_-mediated oxidative cyclization (Scheme 2a) [43]. Tao, Chao, and co-workers succeeded in the preparation of similar tetrasubstituted tetrabenzocoronenes by a Corey–Fuchs reaction, followed by a Suzuki–Miyaura cross-coupling, and a two-step dehydrogenative cyclization using DDQ and FeCl_3_ (Scheme 2b) [44]. However, 3,6,13,16-tetrasubstituted tetrabenzo[*a*,*d*,*j*,*m*]coronenes have not been reported using these reported methods.

Here, we describe a convenient method for the synthesis of 3,6,13,16-tetrasubstituted tetrabenzo[*a*,*d*,*j*,*m*]coronenes based on a ruthenium-catalyzed C–O/C–H multiarylation of anthraquinone and its derivatives.

## Results and Discussion

**Scheme 3:**
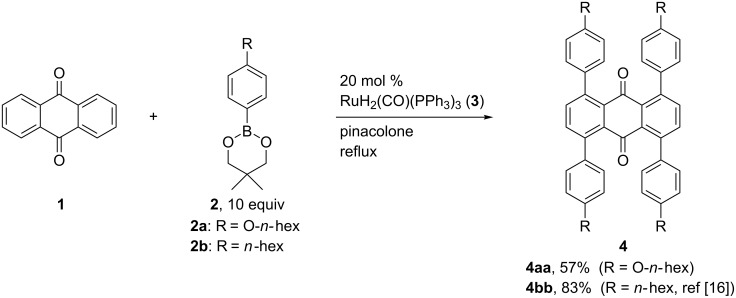
C–H tetraarylation of anthraquinone (**1**).

Our efforts toward the synthesis of tetrabenzo[*a*,*d*,*j*,*m*]coronenes started with the preparation of tetraarylanthraquinones. Previously, we reported the synthesis of anthraquinone derivatives possessing four aryl groups at the 1, 4, 5, and 8-positions through a RuH_2_(CO)(PPh_3_)_3_-catalyzed C–H arylation of anthraquinone (**1**) with arylboronates **2** [16]. Using this method, we examined the synthesis of a tetraarylanthraquinone possessing four hexyloxy groups. When the reaction of **1** with 4-hexyloxyphenylboronate **2a** was carried out in the presence of 20 mol % of RuH_2_(CO)(PPh_3_)_3_ (**3**) in refluxing pinacolone, the corresponding tetraarylation product **4aa** was obtained in 57% yield (Scheme 3). Tetrakis(4-hexylphenyl)anthraquinone (**4bb**), the synthesis of which has been described in the above-mentioned publication [16], was also prepared using 4-hexylphenylboronate (**2b**) accordingly and used for further transformations. The reaction of **1** with **2b** was also attempted using 10 mol % of **3**, however, it only gave mono-, di- and triarylation products, and the tetraarylation product **4bb** was not detected.

**Scheme 4:**
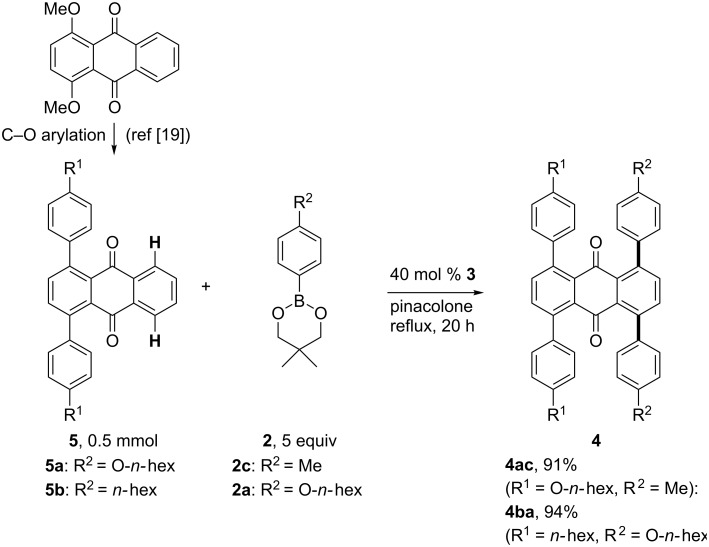
C–H diarylation of 1,4-diarylanthraquinones **5**.

Tetraarylanthraquinones containing two different aryl groups were also prepared via the above-mentioned chemoselective C–O arylation and subsequent C–H arylation. The chemoselective C–O diarylation of 1,4-dimethoxyanthraquinone to form 1,4-diarylanthraquinones has been previously reported by our group [19], and 1,4-bis(4-hexyloxyphenyl)anthraquinone (**5a**) and 1,4-bis(4-hexylphenyl)anthraquinone (**5b**) were prepared accordingly. When the reaction of **5a** with 4-methylphenylboronate **2c** was conducted in the presence of 40 mol % of **3** in refluxing pinacolone, the *ortho*-C–H diarylation occurred giving the 1,4-bis(4-hexyloxyphenyl)-5,8-bis(4-methylphenyl)anthraquinone (**4ac**) in 91% yield. The C–H diarylation of **5b** with **2a** also proceeded smoothly to provide the corresponding tetraarylanthraquinone **4ba** in 94% yield (Scheme 4).

Next, the conversion of tetraarylanthraquinones **4** to tetrabenzo[*a*,*d*,*j*,*m*]coronenes was investigated. After examining various carbonyl methylenation methods, we found that the dimethylenation products **6** can be obtained in high yields through methylation of the carbonyl groups, followed by dehydration. Thus, the reaction of **4aa** with methyllithium and subsequent treatment of the crude diol with NaH_2_PO_2_·H_2_O and NaI in refluxing acetic acid gave the corresponding dimethylenation product **6aa** in 96% yield (Table 1, entry 1). The oxidative cyclization of **6aa** by treatment with 12 equiv of FeCl_3_ at 35 °C for 0.5 h provided the desired tetrabenzo[*a*,*d*,*j*,*m*]corronene derivative **7aa** in 37% yield. This method was also applicable to the conversion of tetrakis(4-hexylphenyl)anthraquinone **4bb** as well as the tetraarylanthraquinones containing two different aryl groups, **4ac** and **4ba**, leading to the corresponding tetrabenzo[*a*,*d*,*j*,*m*]corronene derivatives, **7bb**, **7ac**, and **7ba**, respectively (Table 1, entries 2–4).

**Table 1:** Synthesis of 3,6,13,16-tetrasubstituted tetrabenzo[*a*,*d*,*j*,*m*]coronenes.

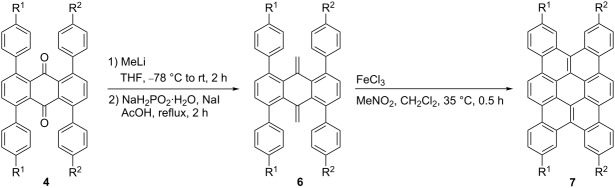				

entry	R^1^	R^2^	yield of **6** (%)^a^	yield of **7** (%)^b^

1	*n*-hexO	*n*-hexO	**6aa**: 96	**7aa**: 37
2	*n*-hex	*n*-hex	**6bb**: 85	**7bb**: 73
3	*n*-hexO	Me	**6ac**: 78	**7ac**: 48
4	*n*-hex	*n*-hexO	**6ba**: 85	**7ba**: 48

^a^Reaction conditions: 1) **4** (0.2 mmol), MeLi (2 mmol), THF (10 mL), −78 °C to rt, 2 h; 2) NaH_2_PO_2_·H_2_O (2 mmol), NaI (2 mmol), AcOH (6 mL), reflux, 2 h; ^b^reaction conditions: **6** (0.02 mmol), FeCl_3_ (0.24 mmol), MeNO_2_ (1 mL), CH_2_Cl_2_ (5 mL), gentle N_2_ bubbling, 35 °C, 0.5 h.

**Figure 1:**
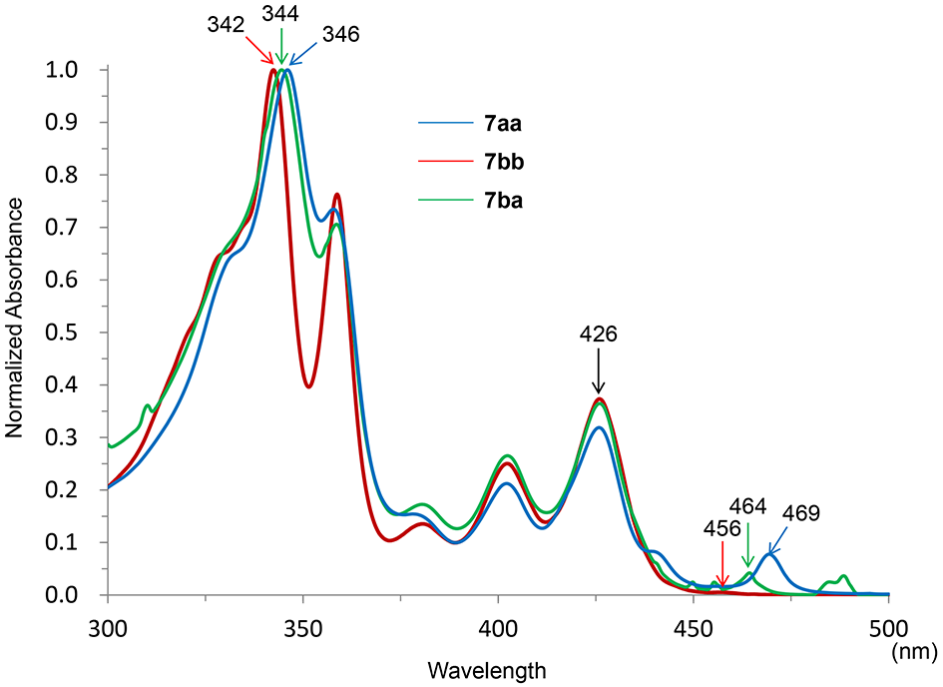
Normalized UV–vis absorption spectra of **7aa**, **7bb**, and **7ba**.

Next, the effect of the substituents in compounds **7** on their optical properties was studied by UV–vis spectroscopy (Figure 1). The UV–vis spectra of **7aa**, **7bb**, and **7ba** were measured in chloroform, and the normalized UV–vis spectra are shown in Figure 1. These compounds showed similar peak patterns between 300 and 500 nm, and the π–π* transitions (p-band) for **7aa**, **7bb**, and **7ba** were observed at 426 nm [11]. The peaks corresponding to n–π* transitions (α-band) were observed at 456–469 nm and were red-shifted with growing intensity as the number of hexyloxy groups increases.

**Figure 2:**
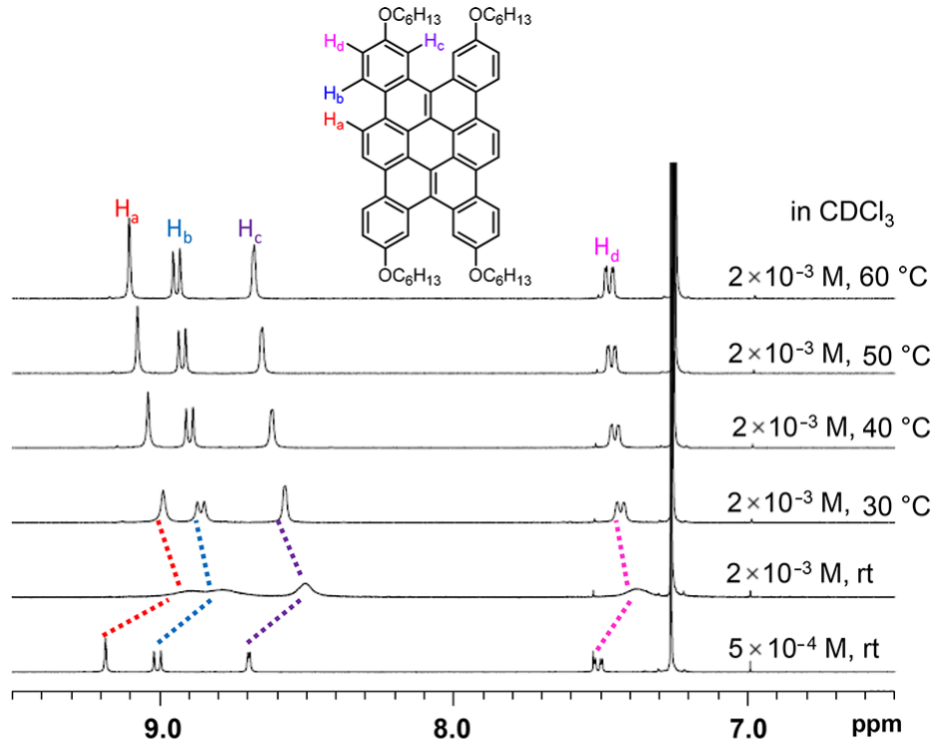
Effects of the concentration and the temperature on the ^1^H NMR spectra of **7aa**.

The self-assembling property of tetrabenzo[*a*,*d*,*j*,*m*]coronene **7aa** was also examined (Figure 2), as Wu and co-workers have studied the self-assembling property of the tetrabenzo[*a*,*d*,*j*,*m*]coronene derivative possessing *n*-octyl groups at the 2, 7, 12, and 17-positions by ^1^H NMR spectroscopy and suggested its intermolecular π–π interaction in solution [10]. When the ^1^H NMR spectrum of **7aa** was measured at 2 × 10^−3^ M at rt, a considerable broadening of the signals in the aromatic region was observed. By increasing the temperature from rt to 60 °C, all signals in the aromatic region became sharper and low-field shifted. The dilution of the solution to 5 × 10^−4^ M at rt also led to a downfield shift and sharpening of the signals. These observations suggest that **7aa** assembles by intermolecular π–π interaction in CDCl_3_ as was seen by Wu and co-workers for their compound possessing substituents at different positions.

## Conclusion

The synthesis of a new class of tetrabenzo[*a*,*d*,*j*,*m*]coronene derivatives having alkyl and alkoxy-substituents at the 3, 6, 13, and 16-positions was achieved based on a ruthenium-catalyzed coupling reaction of anthraquinone derivatives with arylboronates via a C–H or/and C–O bond cleavage. The reaction sequence involving the arylation, carbonyl methylenation, and oxidative cyclization effectively provided various 3,6,13,16-tetrasubstituted tetrabenzo[*a*,*d*,*j*,*m*]coronenes in short steps from readily available starting materials. The tetrabenzo[*a*,*d*,*j*,*m*]coronenes possessing two different types of substituents were obtained selectively by sequential chemoselective C–O and C–H arylations. The strategy developed in this study should be useful for the synthesis of PAH derivatives with multiple substituents having different properties.

## Supporting Information

File 1 General experimental procedures, characterization data and NMR spectra of new compounds.
