# Supplementary material for: Efficient synthesis of 3,6,13,16-tetrasubstituted-tetrabenzo[a,d,j,m]coronenes by selective C–H/C–O arylations of anthraquinone derivatives
Source: Beilstein J Org Chem. 2020 Mar 31;16:544–50. doi: 10.3762/bjoc.16.51 (PMC7136549; doi:10.3762/bjoc.16.51)
Supplement: File 1 — General experimental procedures, characterization data and NMR spectra of new compounds. [file Beilstein_J_Org_Chem-16-544-s001.pdf]

BEILSTEIN JOURNAL OF ORGANIC CHEMISTRY

Supporting Information

for

Efficient synthesis of 3,6,13,16-tetrasubstituted-tetrabenzo[*a*,*d*,*j*,*m*]coronenes by selective C–H/C–O arylations of anthraquinone derivatives

Seiya Terai, Yuki Sato, Takuya Kochi and Fumitoshi Kakiuchi

Beilstein J. Org. Chem. **2020,** *16,* 544–550. doi:10.3762/bjoc.16.51

General experimental procedures, characterization data and NMR spectra of new compounds

License and Terms: This is a supporting information file under the terms of the Creative Commons Attribution License (http://creativecommons.org/licenses/by/4.0). Please note that the reuse, redistribution and reproduction in particular requires that the authors and source are credited.
The license is subject to the *Beilstein Journal of Organic Chemistry* terms and conditions: (https://www.beilstein-journals.org/bjoc)

Table of contents

1. General information S2

2. Preparation of 1,4,5,8-tetraarylanthraquinone **4aa** S2

3. Diarylation of 1,4-diarylanthraquinones S3

4. Carbonyl methylenation of tetraarylanthraquinones **4** S5

5. Oxidative cyclization of 9,10-dimethylene-9,10-dihydroanthracenes **6** S7

6. References S9

7. NMR spectra S10

1. General information

^{1}H and $^{13}C\{^{1}H\}$ NMR spectra were recorded on a JEOL JNM-AL400, JNM-ECX400, or JNM-ECS400 spectrometer. ^{1}H NMR data are reported as follows: chemical shift in ppm (δ), multiplicity (s = singlet, d = doublet, t = triplet, m = multiplet), coupling constant (Hz), relative intensity. ^{13}C NMR data are reported as follows: chemical shift in ppm (δ). IR spectra were recorded on a JASCO Herschel FT/IR-410 infrared spectrometer. Gas chromatography (GC) analyses were performed using a CBP-10 capillary column (25 m × 0.22 mm, film thickness 0.25 μm). GC–MS analyses were performed using a CBP-10 capillary column (25 m × 0.22 mm, film thickness 0.25 μm) with a Shimadzu GCMS-QP2010 gas chromatography mass spectrometer. Flash chromatography was carried out with Kanto Chemical silica gel 60N. Preparative gel-permeation chromatography (GPC) was performed on a Japan Analytical LC-9201 equipped with JAI-GEL 1H/2H. Unless otherwise noted, all reactions were performed under a N_2 atmosphere. Pinacolone was dried over $CaSO_4$ and distilled under nitrogen. Arylboronates **2** [1], $RuH_2(CO)(PPh_3)_3$ (**3**) [2], **4bb** [3], **5a** [1b], and **5b** [1b] were prepared by literature methods.

2. Preparation of 1,4,5,8-tetraarylanthraquinone 4aa

An apparatus consisting of a 5 mL two-necked flask equipped with a reflux condenser, a nitrogen inlet and a magnetic stirring bar, was oven-dried, and then cooled to room temperature under a flow of nitrogen. Anthraquinone (**1**, 21.3 mg, 0.102 mmol), 4-hexyloxyphenylboronic acid 2,2-dimethyl-1,3-propandiol ester (**2a**, 290 mg, 1.00 mmol), and $RuH_2(CO)(PPh_3)_3$ (**3**, 18.8 mg, 0.0205 mmol) were placed in the flask. The flask was evacuated and refilled with nitrogen. This cycle was repeated three times. Pinacolone (0.4 mL) was syringed into the flask. The resulting mixture was refluxed

under nitrogen for 24 h. After cooled to rt, the reaction mixture was mixed with methanol and the resulting precipitate collected by filtration. Purification of the crude material by silica gel column chromatography (eluent: $CHCl_3$) afforded tetraarylanthraquinone **4aa** in 57% yield (52.9 mg, 0.0579 mmol): Mp 241-242 °C; ^{1}H NMR (400 MHz, $CDCl_3$): δ 0.94 (t, J = 6.1 Hz, 12 H), 1.31-1.55 (m, 24 H), 1.77-1.85 (m, 8 H), 3.97 (t, J = 6.5 Hz, 8 H), 6.84 (d, J = 8.8 Hz, 8 H): 7.20 (d, J = 8.8 Hz, 8 H), 7.47 (s, 4 H); ^{13}C NMR (100.5 MHz, $CDCl_3$) δ 14.1, 22.6, 25.8, 29.4, 31.7, 67.9, 113.9, 130.3, 131.7, 134.5, 135.5, 140.3, 158.6, 188.5; IR (KBr): 2931 m, 2867 m, 2357 w, 2333 w, 1670 m, 1608 m, 1576 w, 1516 m, 1458 m, 1374 w, 1318 m, 1247 s, 1213 m, 1179 m, 1126 w, 1025 w, 971 w, 828 m, 637 w, 548 w cm^{-1}. HRMS-ESI (*m*/*z*): [M + Na]$^+$ calcd for $C_{62}H_{72}NaO_6$, 935.5227; found, 935.5226. Anal. Calcd for C, 81.54; H, 7.95; O, 10.51. Found C, 81.46; H, 7.84.

3. Diarylation of 1,4-diarylanthraquinones

General procedure for the synthesis of unsymmetrically substituted 1,4,5,8-tetraarylanthracene-9,10-diones, 4ac and 4ba. An apparatus consisting of a 30 mL two-necked flask equipped with a reflux condenser, a nitrogen inlet, and a magnetic stirring bar was oven-dried, and then cooled to room temperature under a flow of nitrogen. 1,4-Arylanthracene-9,10-dione **5** (0.5 mmol), arylboronic acid 2,2-dimethyl-1,3-propandiol ester (**2**, 2.5 mmol), and $RuH_2(CO)(PPh_3)_3$ (**3**, 0.2 mmol) were placed in the flask. The flask was evacuated and refilled with nitrogen. This cycle was repeated three times. Pinacolone (4 mL) was syringed into the flask. The resulting mixture was refluxed under nitrogen for 20 h and cooled to room temperature. The mixture was extracted with $CHCl_3$ and, after filtration, the mixture was concentrated to dryness. The resulting material was dispersed in MeOH under sonication and filtered. Silica gel column chromatography of the resulting solid (eluent: $CHCl_3$ for **4ac**, hexane/$CHCl_3$ 1:1

for **4ba**) afforded the corresponding 1,4,5,8-tetraarylanthracene-9,10-dione **4**.

1,4-Bis(4-hexyloxyphenyl)-5,8-bis(4-methylphenyl)anthracene-9,10-dione (4ac). Mp 241-242 °C; ^{1}H NMR (392 MHz, $CDCl_3$): δ 0.94 (t, *J* = 7.0 Hz, 6 H), 1.33-1.42 (m, 8 H), 1.47-1.52 (m, 4 H), 1.79-1.86 (m, 4 H), 2.39 (s, 6 H), 3.98 (t, *J* = 6.5 Hz, 4 H), 6.83 (d, *J* = 8.1 Hz, 4 H), 7.14 (d, *J* = 8.1 Hz, 4 H), 7.13-7.21 (m, 8 H), 7.48 (s, 2 H), 7.49 (s, 2 H); ^{13}C NMR (98.5 MHz, $CDCl_3$): δ 14.1, 21.2, 22.6, 25.8, 29.3, 31.6, 67.9, 113.9, 128.6, 128.9, 130.2, 131.8, 134.4, 134.6, 135.4, 135.5, 136.8, 136.9, 140.4, 140.8, 158.6, 188.2; IR (KBr): 2929 m, 2868 m, 1668 m, 1608 m, 1518 s, 1458 m, 1318 m, 1245 s, 1214 s, 1179 m, 1126 m, 1022 m, 971 m, 818 m, 551 w, 509 w cm^{-1}; Anal. calcd for C, 84.29; H, 7.07; O, 8.64. Found C, 84.00; H, 7.05.

1,4-Bis(4-hexyloxyphenyl)-5,8-bis(4-hexylphenyl)anthracene-9,10-dione (4ba). Mp 241-242 °C; ^{1}H NMR (392 MHz, $CDCl_3$): δ 0.92 (t, *J* = 6.8 Hz, 6 H), 0.94 (t, *J* = 6.8 Hz, 6 H), 1.35-1.53 (m, 24 H), 1.67 (tt, *J* = 7.3, 7.3 Hz, 4 H), 1.81 (tt, *J* = 6.8, 6.8 Hz, 4 H), 2.63 (t, *J* = 7.3 Hz, 4 H), 3.96 (t, *J* = 6.8 Hz, 4 H), 6.82 (d, *J* = 8.3 Hz, 4 H), 7.14 (d, *J* = 7.8 Hz, 4 H), 7.18-7.22 (m, 8 H), 7.46 (s, 2 H), 7.48 (s, 2 H); ^{13}C NMR (98.5 MHz, $CDCl_3$): δ 14.06, 14.12, 22.6, 22.7, 25.8, 29.3, 29.4, 31.4, 31.6, 31.7, 35.8, 67.9, 113.8, 127.9, 128.9, 130.2, 131.7, 134.4, 134.5, 135.3, 135.5, 137.0, 140.4, 140.8, 141.9, 158.5, 188.1; IR (KBr) 2955 s, 2928 s, 2855 m, 1670 s, 1608 m, 1517 s, 1458 s, 1374 m, 1319 s, 1244 s, 1213 s, 1178 m, 1126 m, 1021 w, 971 m, 826 s, 760 m, 640 w, 546 m cm^{-1}; HRMS-ESI (*m*/*z*): $[M + Na]^+$ calcd for $C_{62}H_{72}NaO_4$ 903.5328; found, 903.5328; Anal. calcd for C, 84.50; H, 8.24. Found C, 84.39; H, 8.26.

4. Carbonyl methylenation of tetraarylanthraquinones 4

General procedure for the synthesis of 1,4,5,8-tetraryl-9,10-dimethylene-9,10-dihydroanthracenes 6. An apparatus consisting of a 50 mL Schlenk flask equipped with a magnetic stirring bar was oven-dried, and then cooled to room temperature under a flow of nitrogen. 1,4,5,8-Tetraarylanthracene-9,10-dione **4** (0.2 mmol) was placed in the flask. The flask was evacuated and refilled with nitrogen. This cycle was repeated three times. THF (10 mL) and 2 mL of 1 M ether solution of MeLi (2 mmol) were syringed into the flask at −78 °C. The reaction mixture was gradually warmed to rt and stirred for 2 h. Hydrochloric acid (1 M, 12 mL) was added to the mixture, which was then extracted twice with 20 mL of $CHCl_3$. The combined organic layers were washed with 20 mL of $NaHCO_3$ aq (0.1 M), 20 mL of water, and 20 mL of brine, dried over $MgSO_4$, filtered, and concentrated to give the crude diol product. NaI (2 mmol), NaH_2PO_2 (2 mmol), and AcOH (6 mL) were added to the flask containing the crude diol and the mixture was refluxed for 2 h. After cooling to rt, the reaction mixture was diluted with 20 mL of water and extracted twice with $CHCl_3$. The combined organic layers were washed with 20 mL of $NaHCO_3$ aq (0.1 M), 20 mL of water, and 20 mL of brine, dried over $MgSO_4$, filtered, and concentrated. Silica gel column chromatography (eluent: hexane/$CHCl_3$ 1:1 to $CHCl_3$ for **6aa**, hexane/EtOAc 50:1 for **6bb**, hexane/$CHCl_3$ 2:1 for **6ac**, and hexane/EtOAc 20:1 for **6ba**) of the crude material afforded the dimethylenation products **6**.

1,4,5,8-Tetrakis(4-hexyloxyphenyl)-9,10-dimethylene-9,10-dihydroanthracene (6aa). Mp 182-183 °C; ^{1}H NMR (400 MHz, $CDCl_3$): δ 0.93 (t, J = 6.8 Hz, 12 H), 1.31-1.55 (m, 24 H), 1.78-1.85 (m, 8 H), 3.96-4.01 (m, 8 H), 4.89 (s, 4 H), 6.89 (d, J = 8.5 Hz, 8 H), 7.22 (s, 4 H), 7.33 (d, J = 8.5 Hz, 8 H); ^{13}C NMR (100.5 MHz, $CDCl_3$): δ 14.1, 22.6, 25.8, 29.4, 31.7, 67.9, 113.9, 124.4, 130.0, 130.6, 134.3, 135.8, 137.9, 139.0, 157.9; IR (KBr): 2931 s, 2861 m, 2360 w, 2331 w, 1611 m, 1577 w, 1517 s, 1461 m, 1390 w, 1283 m, 1243

s, 1176 m, 1107 w, 1031 w, 911 w, 823 m, 538 w cm^{-1}; HRMS-ESI (*m*/*z*): [M + Na]$^+$ calcd for $C_{64}H_{76}NaO_4$ 931.5641; found, 931.5609; Anal. calcd for C, 84.54; H, 8.42. Found: C, 84.46; H, 8.37

1,4,5,8-Tetrakis(4-hexylphenyl)-9,10-dimethylene-9,10-dihydroanthracene (6bb). Mp 182-183 °C; ^{1}H NMR (392 MHz, $CDCl_3$): δ 0.91 (t, *J* = 7.0 Hz, 12 H), 1.33-1.42 (m, 24 H), 1.63-1.70 (m, 8 H), 2.61-2.65 (m, 8 H), 4.84 (s, 4 H), 7.17 (d, *J* = 8.1 Hz, 8 H), 7.25 (s, 4 H), 7.35 (d, *J* = 8.1 Hz, 8 H); ^{13}C NMR (98.5 MHz, $CDCl_3$): δ14.1, 22.7, 29.2, 31.6, 31.8, 35.8, 124.7, 127.9, 129.5, 129.9, 135.8, 138.3, 138.9, 139.4, 141.1; IR (KBr) 3023 w, 2958 s, 2925 s, 2853 s, 2359 w, 1903 w, 1620 w, 1517 w, 1463 s, 1377 w, 1318 w, 1258 w, 1181 w, 1115 w, 1019 w, 917 m, 818 m,698 w, 536 w cm^{-1}; HRMS-ESI (*m*/*z*): [M + Na]$^+$ calcd for $C_{64}H_{76}Na$ 867.5845; found, 867.5848; Anal. calcd for C, 90.94 H, 9.06. Found C, 90.74, H, 9.00.

1,4-Bis(4-hexyloxyphenyl)-9,10-dimethylene-5,8-bis(4-methylphenyl)-9,10-dihydroanthracene (6ac). Mp 189-190 °C; ^{1}H NMR (392 MHz, $CDCl_3$): δ 0.93 (t, *J* = 6.7 Hz, 6 H), 1.37-1.40 (m, 8 H), 1.47-1.53 (m, 4 H), 1.78-1.87 (m, 4 H), 2.40 (s, 6 H), 3.99 (dt, *J* = 6.5, 6.5 Hz, 4 H), 4.86 (s, 2 H), 4.89 (s, 2 H), 6.87 (d, *J* = 8.5 Hz, 4 H), 7.18 (d, *J* = 7.6 Hz, 4 H), 7.23-7.24 (m, 4 H), 7.32-7.34 (m, 8H); ^{13}C NMR (98.5 MHz, $CDCl_3$): δ 14.1, 21.3, 22.6, 25.8, 29.3, 31.6, 68.0, 113.9, 124.6, 128.7 129.6, 129.9, 130.0, 130.6, 134.3, 135.75, 135.82, 136.0, 137.9, 138.2, 139.0, 139.3, 157.9; IR (KBr): 2951 m, 2930 m, 2869 w, 1611 m, 1519 m, 1464 s, 1388 w, 1286 w, 1242 s, 1174 m, 1105 w, 1054 w, 1021 w, 910 m, 839 w, 814 m cm^{-1}; HRMS-ESI (*m*/*z*): [M + Na]$^+$ calcd for $C_{54}H_{56}O_2Na$ 759.4178; found, 759.4206; Anal. calcd for C, 88.00; H, 7.66. Found: C, 87.75; H, 7.65.

1,4-Bis(4-hexyloxyphenyl)-5,8-bis(4-hexylphenyl)-9,10-dimethylene-9,10-dihydroanthracene (6ba). Mp 168-169 °C; ^{1}H NMR (392 MHz, $CDCl_3$): δ 0.90 (t, *J* = 7.2 Hz, 6 H), 0.94 (t, *J* = 7.2 Hz, 6 H), 1.33-1.42 (m, 20 H), 1.47-1.54 (m, 4 H), 1.63-1.71 (m, 4 H), 1.79-1.86 (m, 4 H), 2.62-2.66 (m, 4 H), 3.98 (dt, *J* = 6.3, 6.3 Hz, 4 H), 4.86 (d, *J* = 0.9 Hz, 2 H), 4.88 (d, *J* = 0.9 Hz, 2 H), 6.87 (d, *J* = 8.8 Hz, 4 H), 7.18 (d, *J* = 8.1 Hz, 4 H),

7.23 (s, 2 H), 7.24 (s, 2 H), 7.32-7.36 (m, 8 H); ^{13}C NMR (98.5 MHz, $CDCl_3$): δ 14.07, 14.11, 22.65, 22.65, 25.9, 29.2, 29.4, 31.5, 31.7, 31.8, 35.8, 67.9, 113.9, 124.6, 127.9, 129.5, 129.9, 130.0, 130.6, 134.3, 135.76, 135.82, 137.9, 138.3, 138.9, 139.5, 141.1, 157.9; IR (KBr): 2955 s, 2925 s, 2854 s, 1610 m, 1577 w, 1516 s, 1461 s, 1390 w, 1285 m, 1241 s, 1172 m, 1107 w, 1031 w, 909 m, 819 s, 699 w, 668 w, 560 w, 535 w cm^{-1}; HRMS-ESI (*m*/*z*): $[M + Na]^+$ calcd for $C_{64}H_{76}O_2Na$ 899.5743; found, 899.5739; Anal. calcd for C, 87.62; H, 8.73. Found: C, 87.37; H, 8.75.

5. Oxidative cyclization of 9,10-dimethylene-9,10-dihydroanthracenes 6

General procedure for the synthesis of 3,6,13,16-tetrasubstituted tetrabenzo[*a*,*d*,*j*,*m*]coronenes 7. To an apparatus consisting of an oven-dried 20 mL two-necked flask equipped with a rubber septum, an inlet tube sealed with a rubber septum, and a magnetic stirring bar was added 1,4,5,8-tetraryl-9,10-dimethylene-9,10-dihydroanthracene **6** (0.020 mmol). The flask was evacuated and refilled with nitrogen. This cycle was repeated three times. Dichloromethane (5 mL) and a nitromethane (1 mL) solution of $FeCl_3$ (0.24 mmol) were syringed into the flask. Dry nitrogen was slowly passed through the reaction mixture with heating at 35 °C for 30 min. The reaction mixture was cooled to room temperature and then mixed with 10 mL of MeOH and 20 mL of $NaHCO_3$ aq (0.1 M) in this order. The aqueous layer was extracted twice with 20 mL of $CHCl_3$. The combined organic layers were washed with 20 mL of water and 20 mL of brine, dried over Na_2SO_4, filtered, and concentrated. Reprecipitation from hot $CHCl_3$ or toluene/hexane afforded the tetrabenzo[*a*,*d*,*j*,*m*]coronenes **7**. Further purification of the filtrate by gel permeation chromatography also provided **7**.

3,6,13,16-Tetrahexyloxytetrabenzo[*a*,*d*,*j*,*m*]coronene (7aa). ^{1}H NMR (392 MHz, $CDCl_3$): δ 0.95 (t, J = 7.0 Hz, 12 H), 1.36-1.47 (m, 16 H), 1.55-1.63 (m, 8 H), 1.90-1.97 (m, 8 H), 4.23 (t, J = 6.5 Hz, 8 H), 7.47 (dd, J = 9.2, 2.0 Hz, 4 H), 8.68 (d, J = 2.0 Hz, 4 H),

8.94 (d, *J* = 9.2 Hz, 4 H), 9.10 (s, 4 H); ^{13}C NMR (98.5 MHz, $CDCl_3$): *δ* 14.0, 22.7, 26.0, 29.7, 31.8, 68.7, 111.0, 117.0, 121.4, 122.4, 123.0, 123.7, 124.7, 125.2, 126.9, 130.0, 157.3; IR (KBr): 2937 s, 2856 m, 1621 s, 1544 w, 1467 s, 1390 w, 1304 w, 1245 s, 1200 s, 1087 m, 1033 m, 793 m cm^{-1}; MALDI-MS calcd for $[M]^+$ $C_{64}H_{68}O_4$ 900.51; Found 900.64.

3,6,13,16-Tetrahexyltetrabenzo[*a*,*d*,*j*,*m*]coronene (7bb). ^1H NMR (392 MHz, $CDCl_3$): *δ* 0.92 (t, *J* = 7.0 Hz, 12 H), 1.33-1.45 (m, 16 H), 1.49-1.57 (m, 8 H), 1.90 (tt, *J* = 7.6, 7.6 Hz, 8 H), 2.96 (t, *J* = 7.6 Hz, 8 H), 7.68 (d, *J* = 8.7 Hz, 4 H), 8.98 (d, *J* = 8.7 Hz, 4 H), 8.99 (s, 4 H), 9.20 (s, 4 H); IR (KBr): 3086 w, 2957 m, 2923 s, 2851 s, 1616 w, 1467 m, 1388 w, 1253 w, 944 w, 889 w, 805 w, 716 w, 669 w, 560 w cm^{-1}. MALDI- MS calcd for $[M]^+$ $C_{64}H_{68}$ 836.53; Found 836.54.

3,16-Dihexyloxy-6,13-dimethyltetrabenzo[*a*,*d*,*j*,*m*]coronene (7ac). ^1H NMR (392 MHz, $CDCl_3$): *δ* 0.95 (t, *J* = 7.0 Hz, 6 H), 1.41-1.47 (m, 8 H), 1.57-1.64 (m, 4 H), 1.93-2.01 (m, 4 H), 2.71 (s, 6 H), 4.25 (t, *J* = 6.5 Hz, 4 H), 7.49 (dd, *J* = 8.8, 2.0 Hz, 2 H), 7.67 (d, *J* = 8.8 Hz, 2 H), 8.64 (d, *J* = 2.0 Hz, 2 H), 8.95 (s, 2 H), 8.98 (s, 2 H), 9.08 (s, 2 H), 9.14 (s, 2H), 9.21 (s, 2/H); IR (KBr): 2923 s, 2860 s, 1725 w, 1613 s, 1540 m, 1469 s, 1381 w, 1296 w, 1254m, 1233 s, 1206 s, 1112 w, 1080 m, 1034 m, 796 s cm^{-1}; MALDI-MS calcd for $[M+H]^+$ $C_{54}H_{49}O_2$ 729.33; Found 729.55.

3,16-Dihexyl-6,13-dihexyloxytetrabenzo[*a*,*d*,*j*,*m*]coronene (7ba). ^1H NMR (400 MHz, $CDCl_3$) *δ* 0.92 (t, *J* = 6.7 Hz, 6H), 0.96 (t, *J* = 7.0 Hz, 6H), 1.36-1.44 (m, 16 H), 1.46-1.54 (m, 4 H), 1.60 (tt, *J* = 7.4, 7.4 Hz, 4 H), 1.86 (tt, *J* = 7.6, 7.6 Hz, 4 H), 1.90-1.98 (m, 4 H), 2.91 (t, *J* = 7.6 Hz, 4 H), 4.21 (t, *J* = 6.5 Hz, 4 H), 7.41 (dd, *J* = 6.5, 2.2 Hz, 2 H), 7.60 (d, *J* = 8.8 Hz, 2 H), 8.53 (d, *J* = 2.2 Hz, 2 H), 8.81-8-85 (m, 4 H), 8.95 (s, 2H), 8.96 (s, 2H), 9.01 (s, 2H); ^{13}C NMR (100 MHz, $CDCl_3$) *δ* 13.97, 14.02, 22.67, 22.68, 26.0, 29.4, 29.6, 31.7, 31.9, 32.0, 36.7, 68.6, 111.6, 117.3, 121.6, 121.7, 122.6, 123.4, 123.5, 123.6, 123.7, 124.9, 125.1, 127.1, 127.4, 127.5, 128.1, 128.5, 129.0, 130.1, 140.4, 157.4; IR (KBr): 2956 s, 2923 s, 2854 s, 1731 m, 1617 m, 1541 w, 1467

m, 1385 w, 1286 m, 1259 m, 1231 m, 1203 s, 1117 m, 1077 m, 861 w, 798 m cm^{-1}; MALDI-MS calcd for $[M+H]^+$ $C_{64}H_{69}O_2$ 869.53; Found 869.58.

6. References

1. (a) Kondo, H.; Akiba, N.; Kochi, T.; Kakiuchi, F. *Angew. Chem. Int. Ed.* **2015**, *54*, 9293-9297. (b) Suzuki, Y.; Yamada, K.; Watanabe, K.; Kochi, T.; Ie, Y.; Aso, Y.; Kakiuchi, F. *Org. Lett.* **2017**, *19*, 3791-3794.
2. Kakiuchi, F.; Sekine, S.; Tanaka, Y.; Kamatani, A.; Sonoda, M.; Chatani, N.; Murai, S. *Bull. Chem. Soc. Jpn*. **1995**, *68*, 62-83.
3. Kitazawa, K.; Kochi, T.; Sato, M.; Kakiuchi, F. *Org. Lett.* **2009**, *11*, 1951-195.

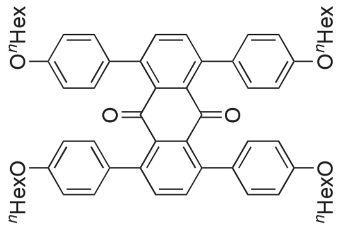

4aa

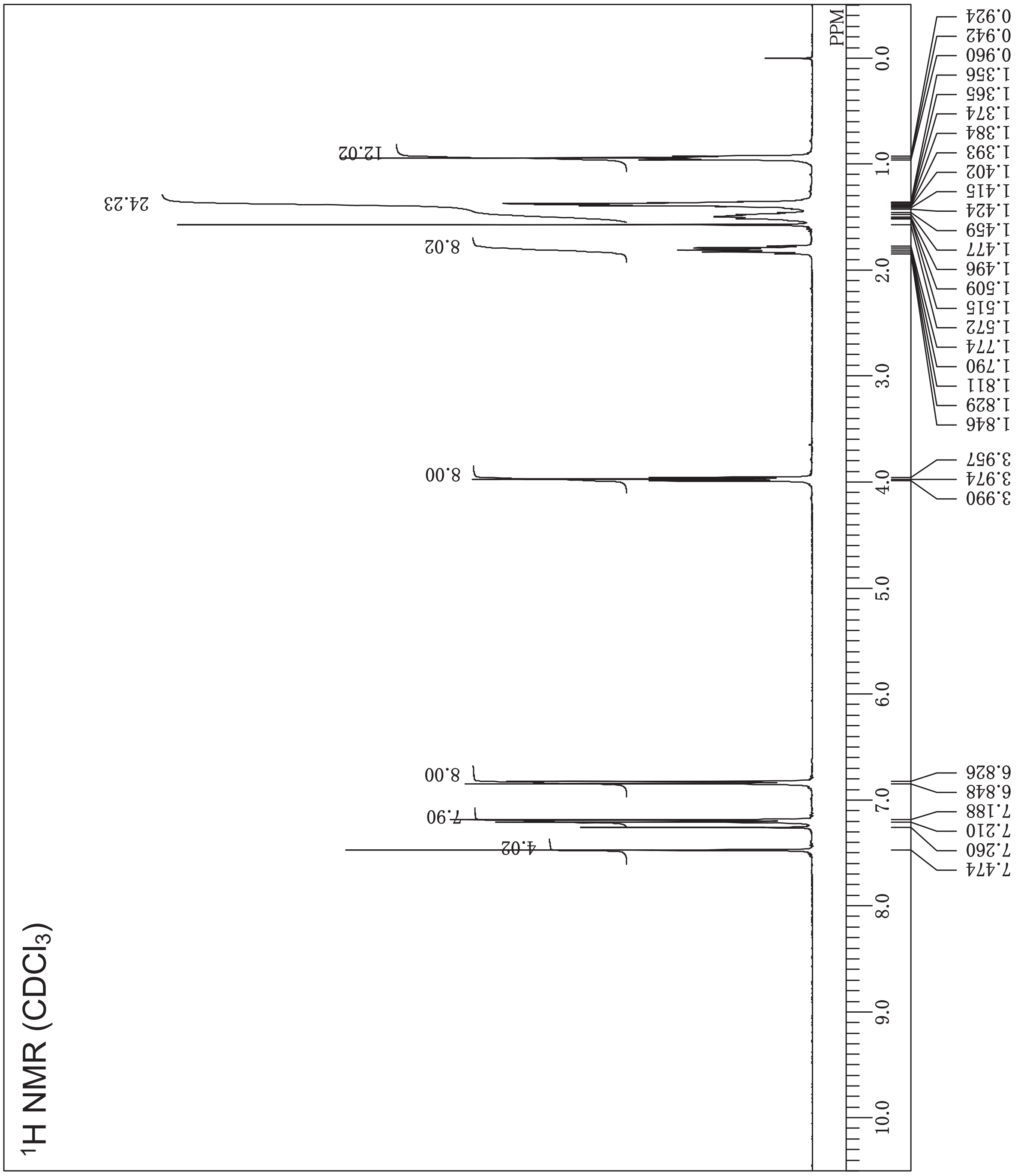

1H NMR (CDCl3)
PPM
0.0
1.0
2.0
3.0
4.0
5.0
6.0
7.0
8.0
9.0
10.0
12.02
24.23
8.02
8.00
8.00
7.90
4.02
0.924
0.942
0.960
1.356
1.365
1.374
1.384
1.393
1.402
1.415
1.424
1.459
1.477
1.496
1.509
1.515
1.572
1.774
1.790
1.811
1.829
1.846
3.957
3.974
3.990
6.826
6.848
7.188
7.210
7.260
7.474

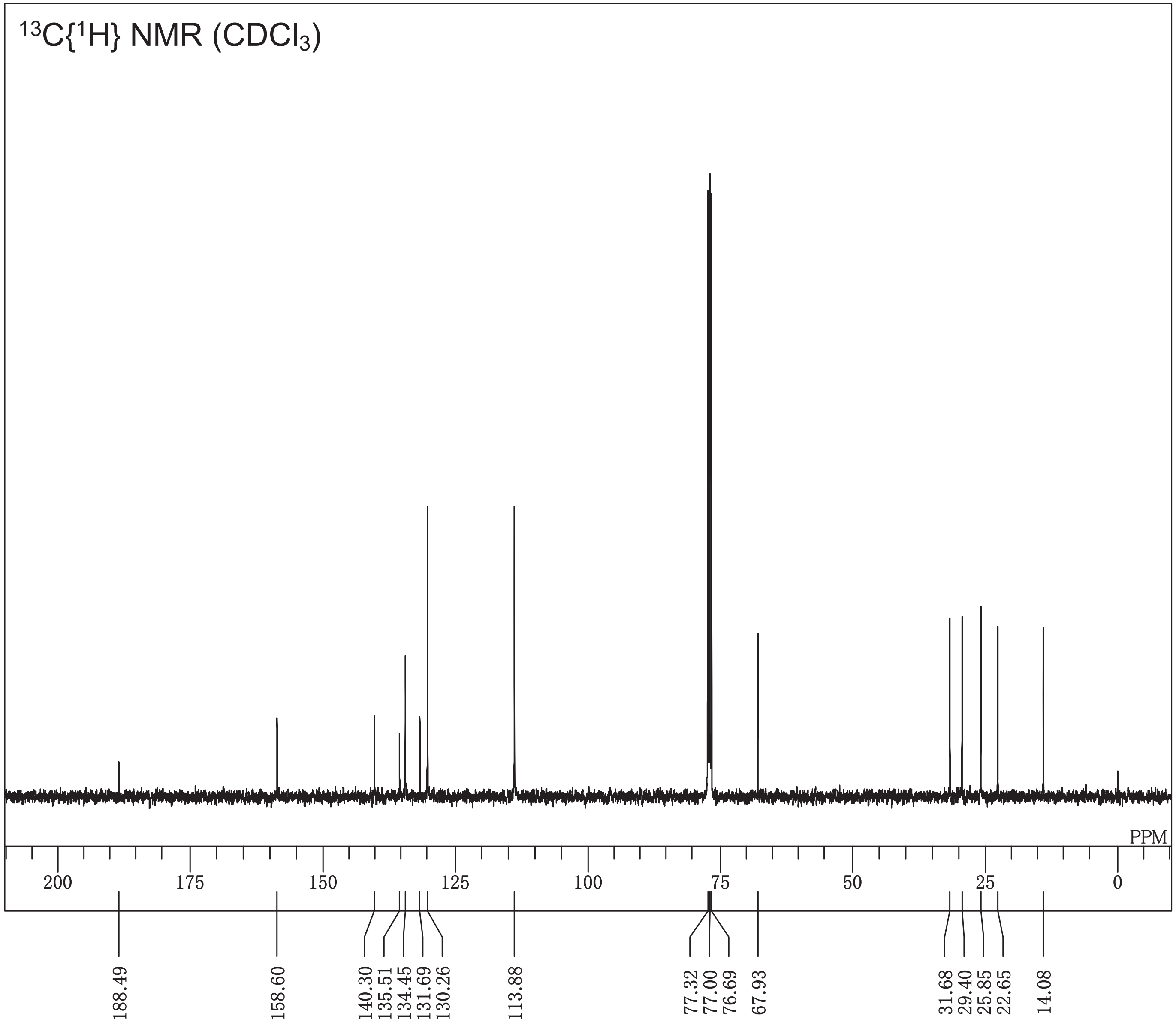
13C{1H} NMR (CDCl3)
PPM
200
175
150
125
100
75
50
25
0
188.49
158.60
140.30
135.51
134.45
131.69
130.26
113.88
77.32
77.00
76.69
67.93
31.68
29.40
25.85
22.65
14.08

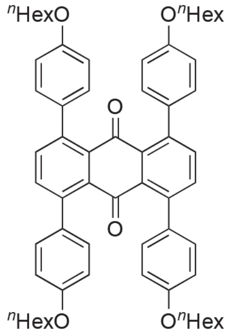
nHexO
OnHex
O
O
nHexO
OnHex

4aa

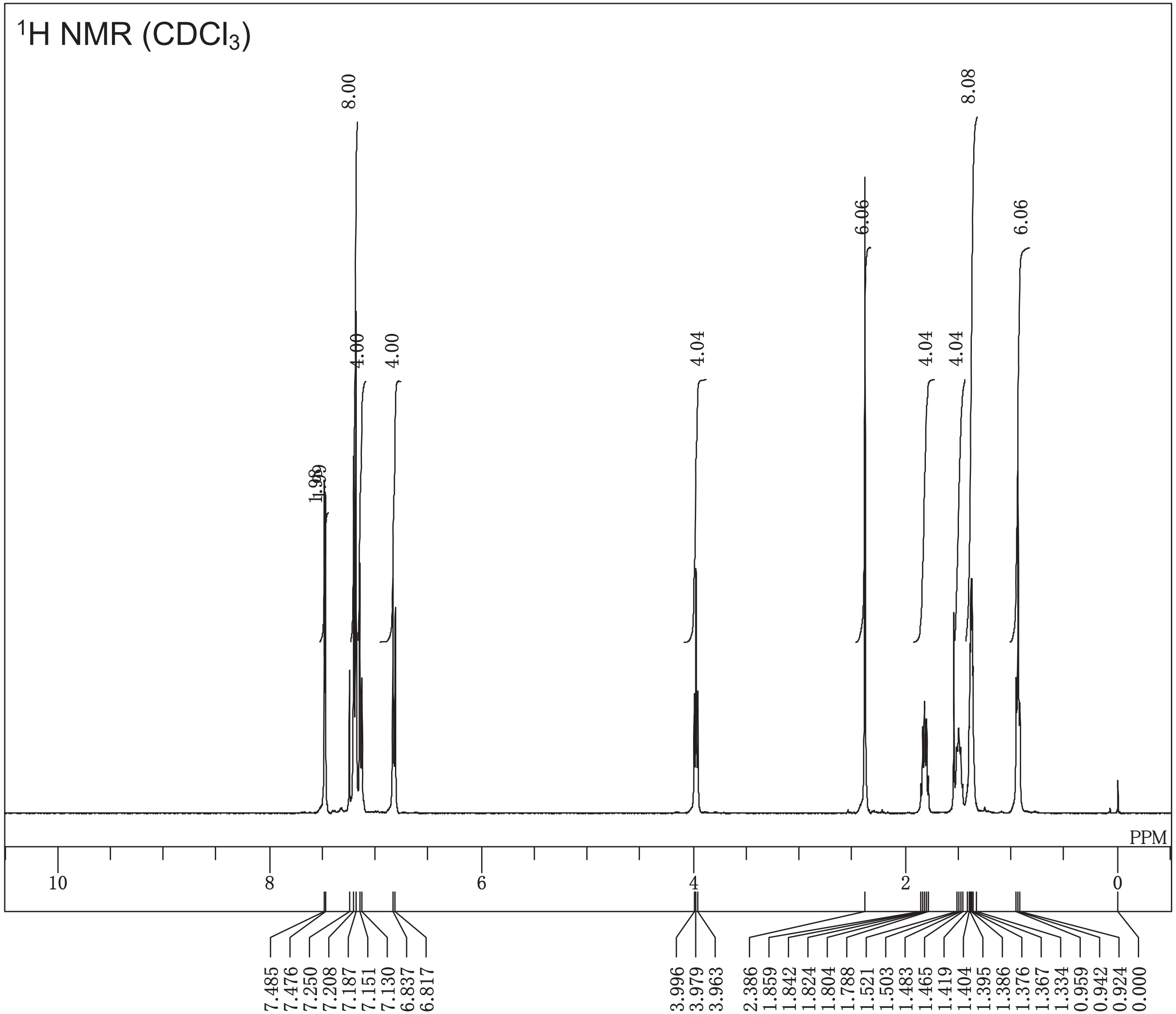

1H NMR (CDCl3)
8.00
4.00
4.00
1.98
1.99
4.04
6.06
4.04
4.04
8.08
6.06
PPM
10
8
6
4
2
0
7.485
7.476
7.250
7.208
7.187
7.151
7.130
6.837
6.817
3.996
3.979
3.963
2.386
1.859
1.842
1.824
1.804
1.788
1.521
1.503
1.483
1.465
1.419
1.404
1.395
1.386
1.376
1.367
1.334
0.959
0.942
0.924
0.000

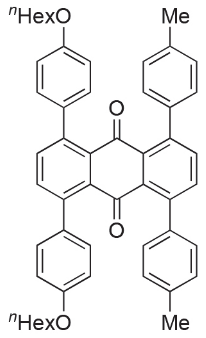

nHexO
Me
O
O
nHexO
Me

4ac

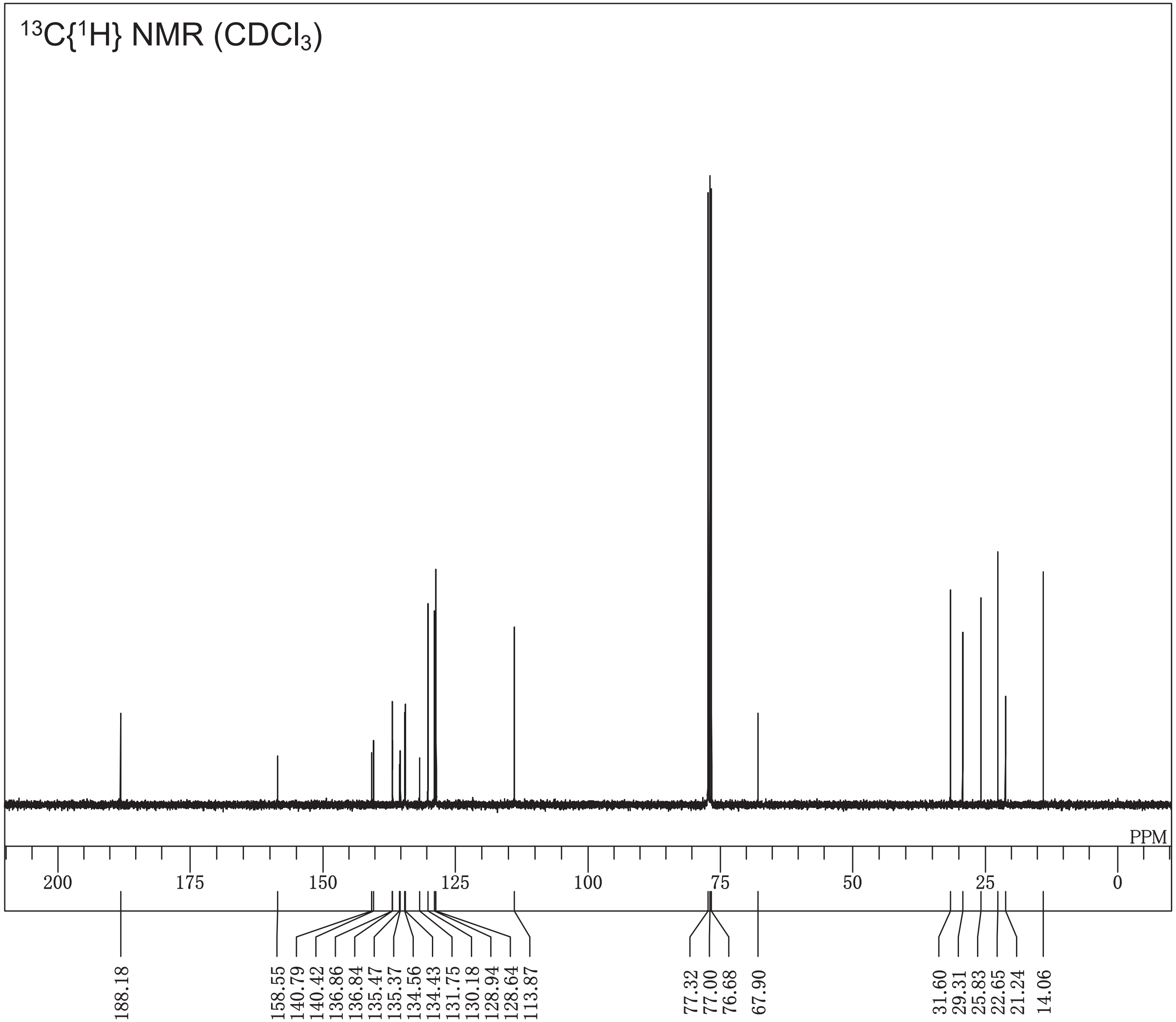

$^{13}C\{^{1}H\}$ NMR ($CDCl_3$)
PPM
200
175
150
125
100
75
50
25
0
188.18
158.55
140.79
140.42
136.86
136.84
135.47
135.37
134.56
134.43
131.75
130.18
128.94
128.64
113.87
77.32
77.00
76.68
67.90
31.60
29.31
25.83
22.65
21.24
14.06

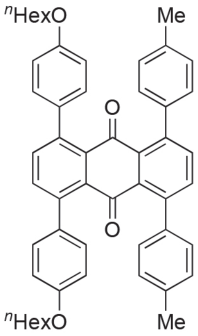

^{n}HexO
Me
O
O
^{n}HexO
Me

4ac

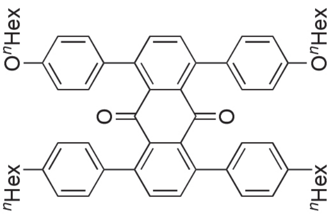
O^{n}Hex
O^{n}Hex
^{n}Hex
^{n}Hex

4ba

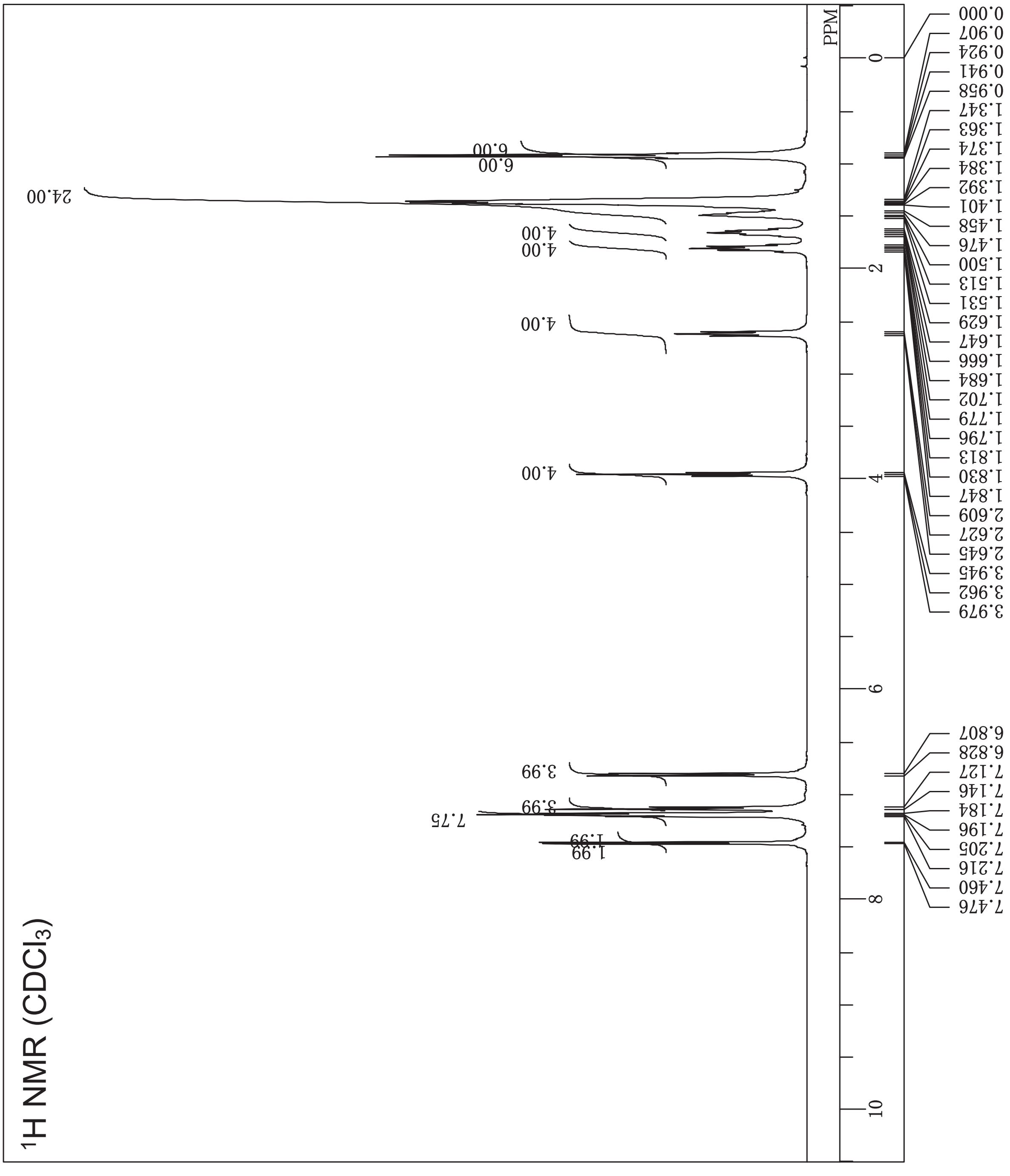
^{1}H NMR ($CDCl_3$)
PPM
0
2
4
6
8
10
24.00
6.00
6.00
4.00
4.00
4.00
4.00
3.99
3.99
7.75
1.99
1.99
0.000
0.907
0.924
0.941
0.958
1.347
1.363
1.374
1.384
1.392
1.401
1.458
1.476
1.500
1.513
1.531
1.629
1.647
1.666
1.684
1.702
1.779
1.796
1.813
1.830
1.847
2.609
2.627
2.645
3.945
3.962
3.979
6.807
6.828
7.127
7.146
7.184
7.196
7.205
7.216
7.460
7.476

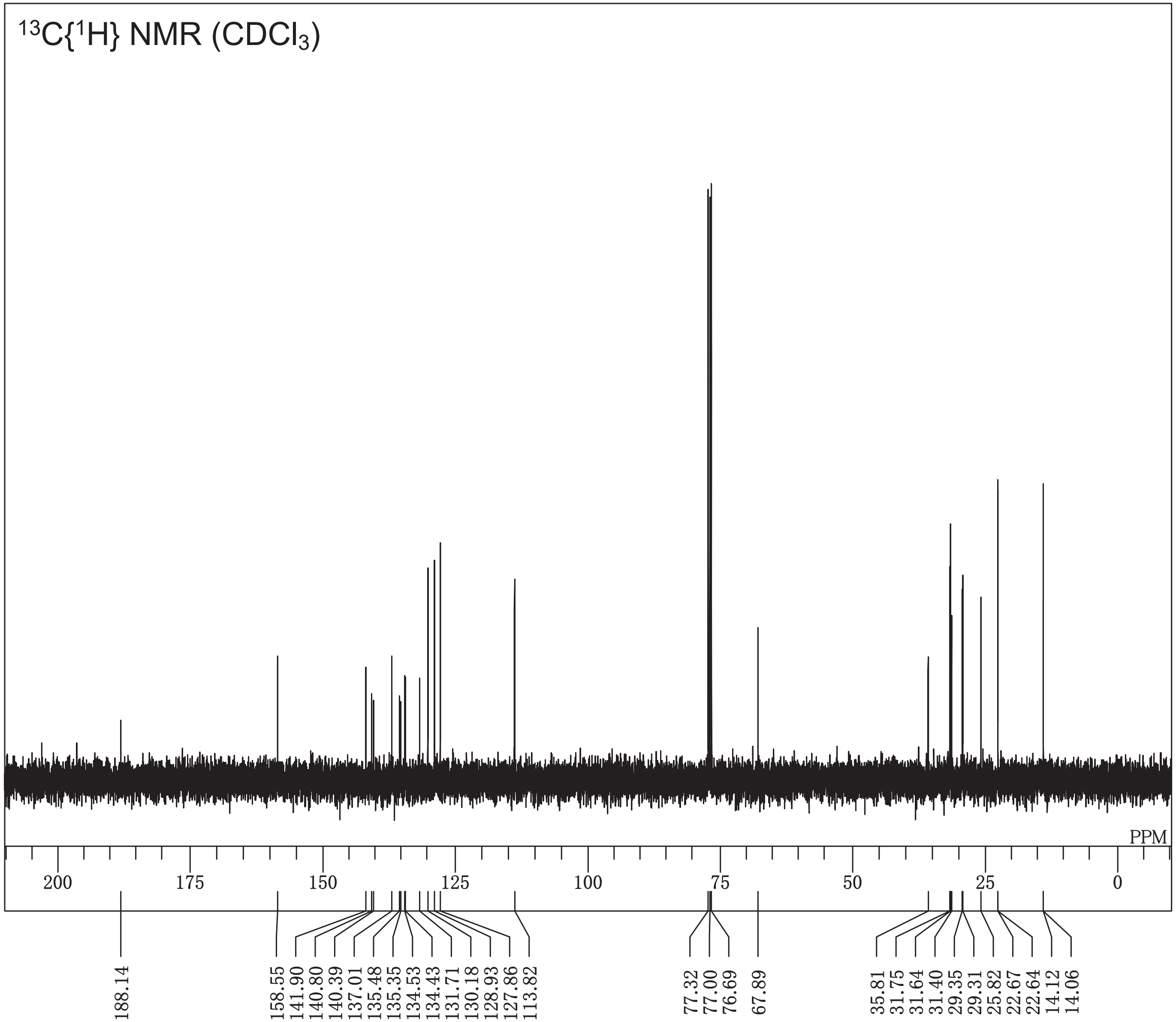

$^{13}C\{^1H\}$ NMR ($CDCl_3$)
PPM
200
175
150
125
100
75
50
25
0
188.14
158.55
141.90
140.80
140.39
137.01
135.48
135.35
134.53
134.43
131.71
130.18
128.93
127.86
113.82
77.32
77.00
76.69
67.89
35.81
31.75
31.64
31.40
29.35
29.31
25.82
22.67
22.64
14.12
14.06

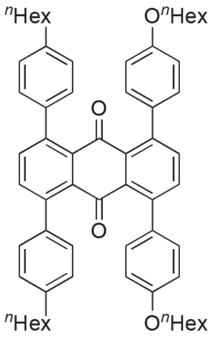

^{n}Hex
O^{n}Hex
O
O
^{n}Hex
O^{n}Hex

4ba

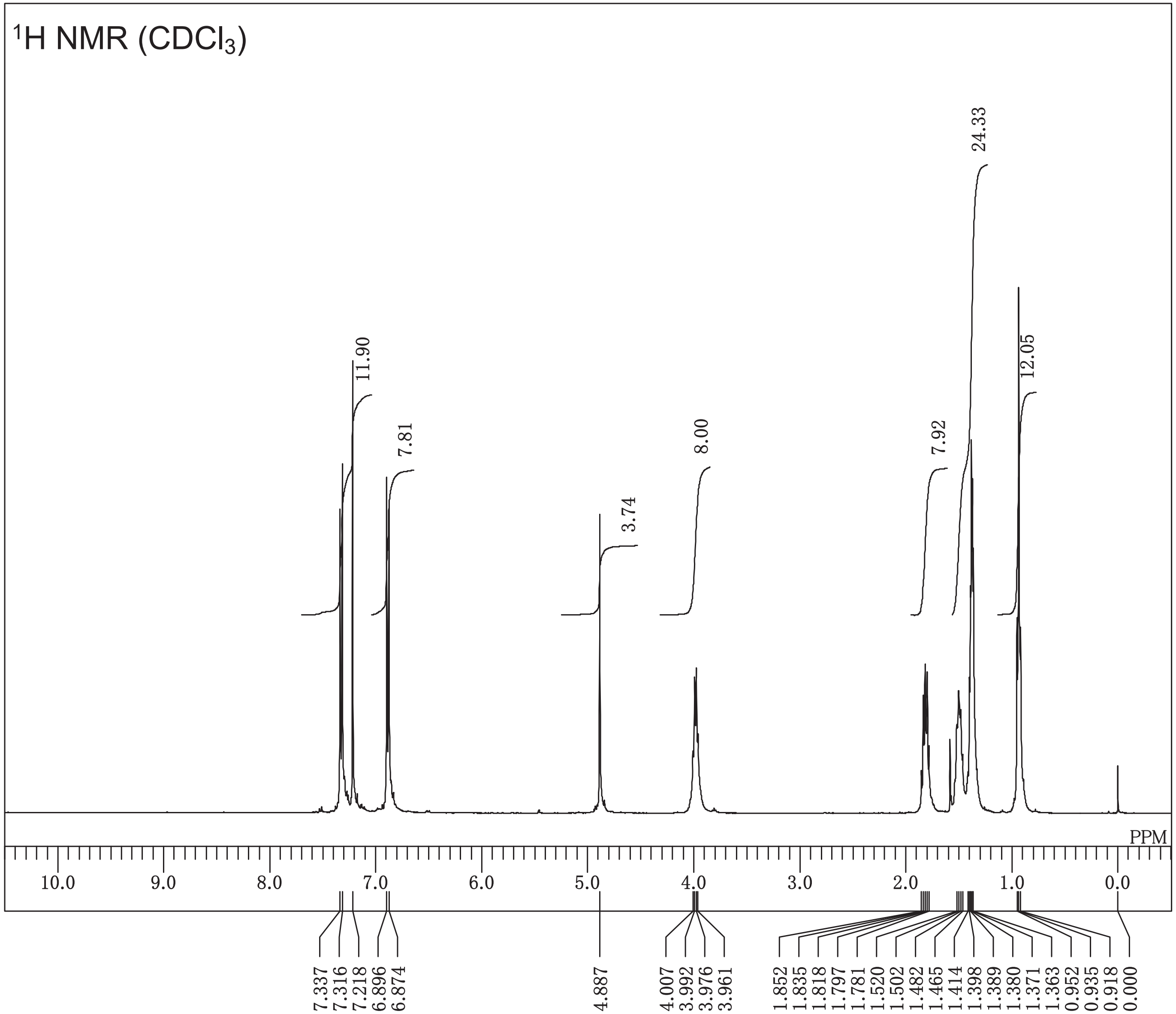

^{1}H NMR ($CDCl_3$)
11.90
7.81
3.74
8.00
7.92
24.33
12.05
PPM
10.0
9.0
8.0
7.0
6.0
5.0
4.0
3.0
2.0
1.0
0.0
7.337
7.316
7.218
6.896
6.874
4.887
4.007
3.992
3.976
3.961
1.852
1.835
1.818
1.797
1.781
1.520
1.502
1.482
1.465
1.414
1.398
1.389
1.380
1.371
1.363
0.952
0.935
0.918
0.000

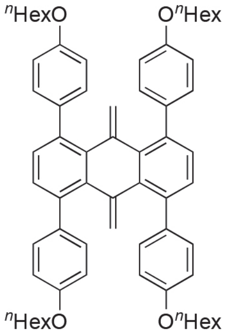

^{n}HexO
O^{n}Hex
^{n}HexO
O^{n}Hex

6aa

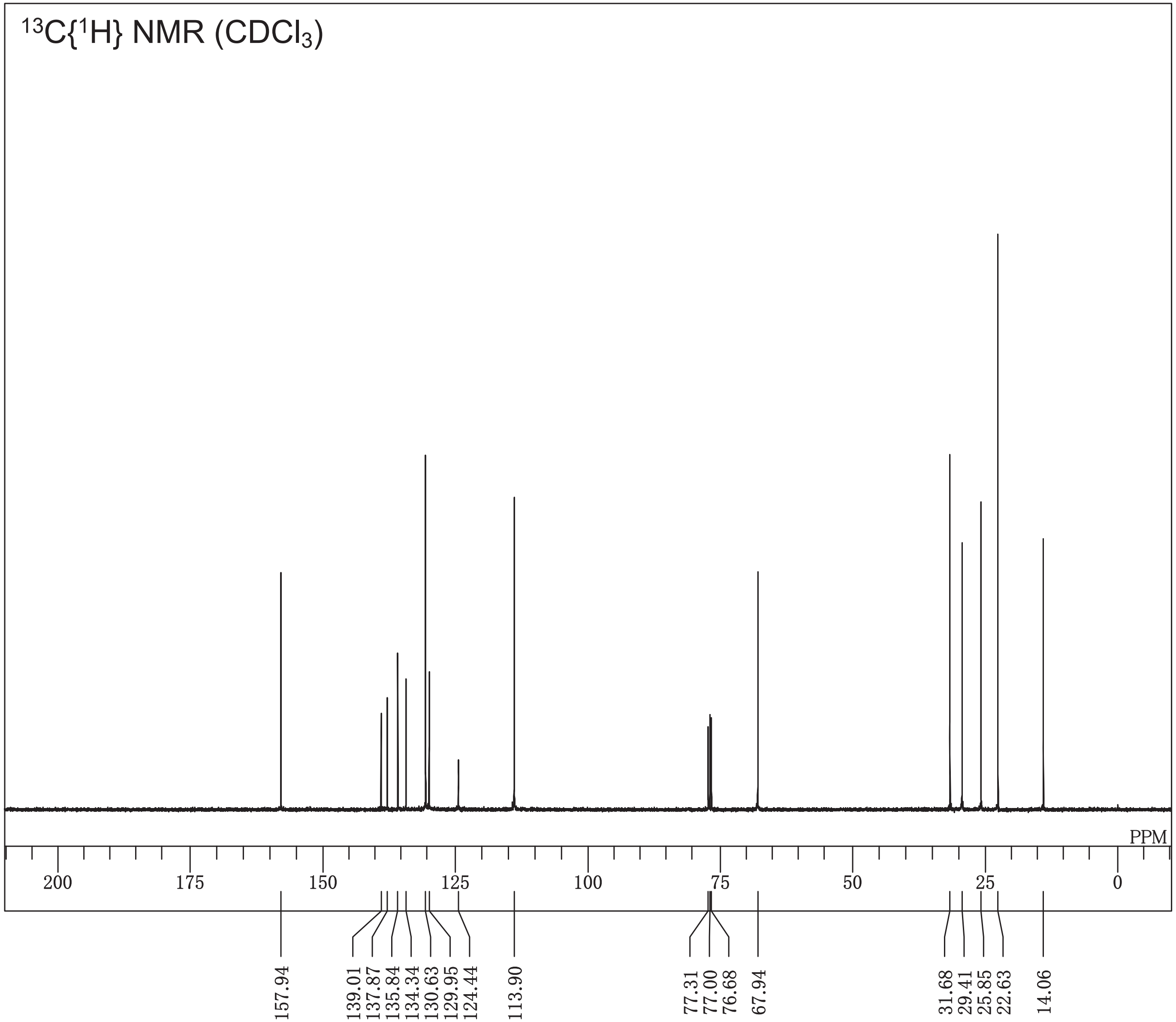

$^{13}C\{^1H\}$ NMR ($CDCl_3$)
PPM
200
175
150
125
100
75
50
25
0
157.94
139.01
137.87
135.84
134.34
130.63
129.95
124.44
113.90
77.31
77.00
76.68
67.94
31.68
29.41
25.85
22.63
14.06

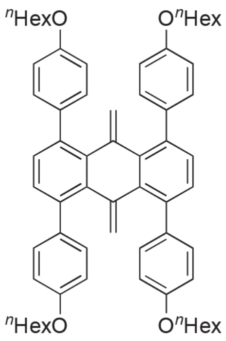

^{n}HexO
O^{n}Hex
^{n}HexO
O^{n}Hex

6aa

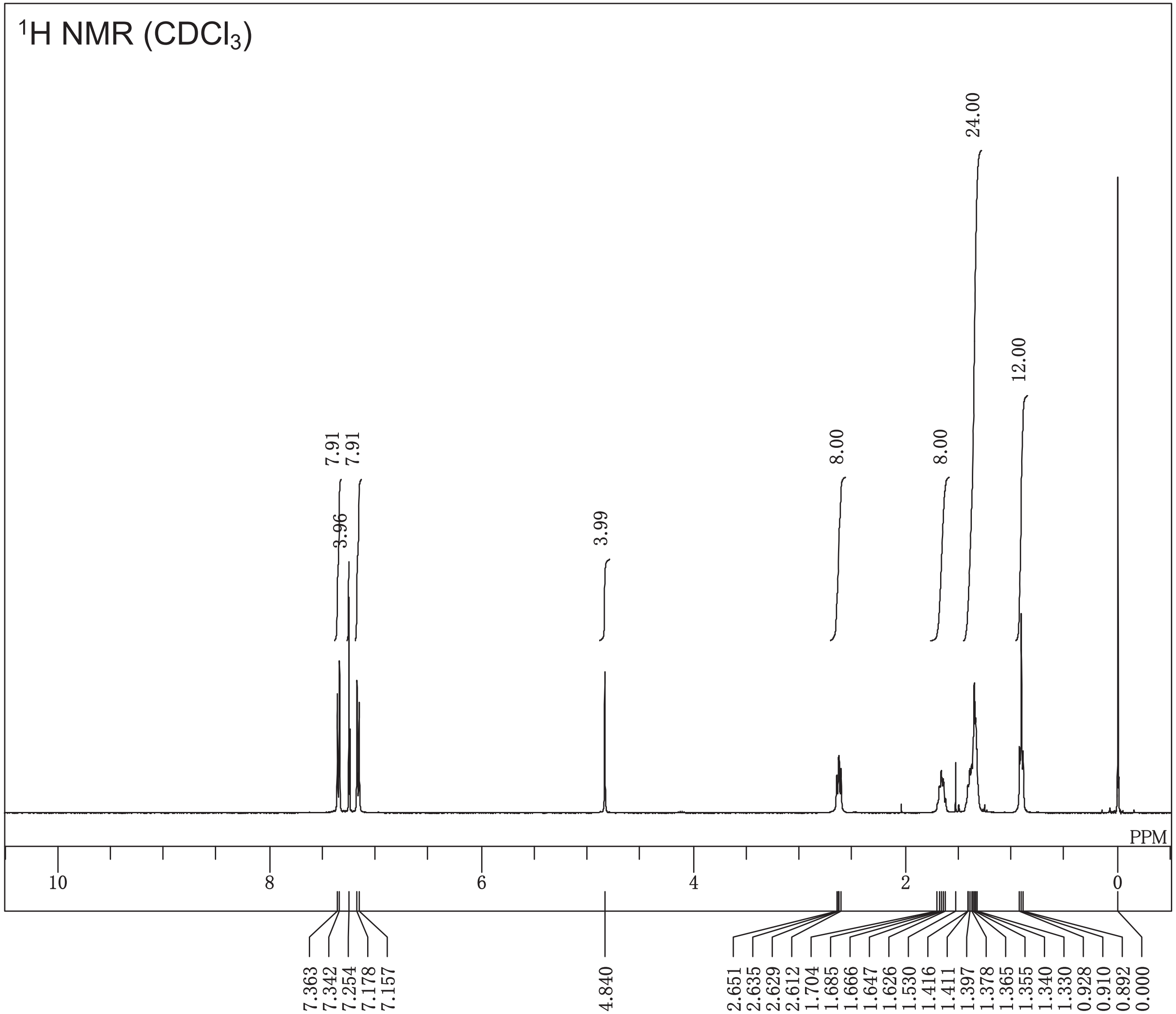

1H NMR (CDCl3)
7.91
7.91
3.96
3.99
8.00
8.00
24.00
12.00
PPM
10
8
6
4
2
0
7.363
7.342
7.254
7.178
7.157
4.840
2.651
2.635
2.629
2.612
1.704
1.685
1.666
1.647
1.626
1.530
1.416
1.411
1.397
1.378
1.365
1.355
1.340
1.330
0.928
0.910
0.892
0.000

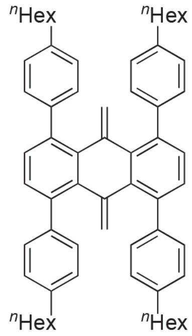

nHex
nHex
nHex
nHex

6bb

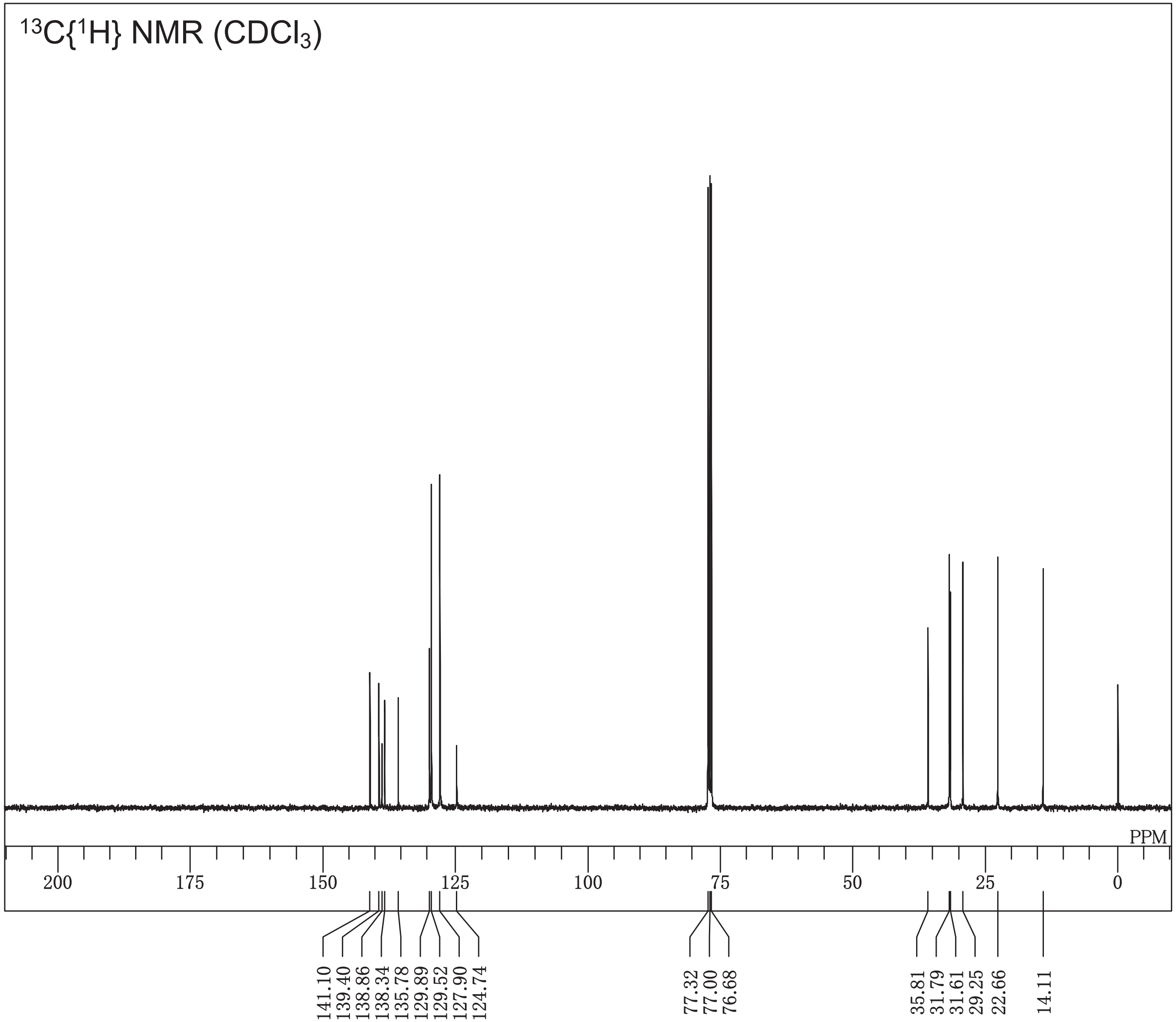

$^{13}C\{^1H\}$ NMR ($CDCl_3$)
PPM
200
175
150
125
100
75
50
25
0
141.10
139.40
138.86
138.34
135.78
129.89
129.52
127.90
124.74
77.32
77.00
76.68
35.81
31.79
31.61
29.25
22.66
14.11

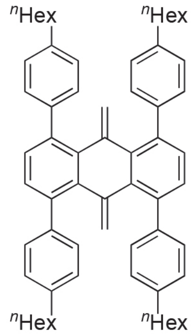

^{n}Hex
^{n}Hex
^{n}Hex
^{n}Hex

6bb

^1H NMR ($CDCl_3$)

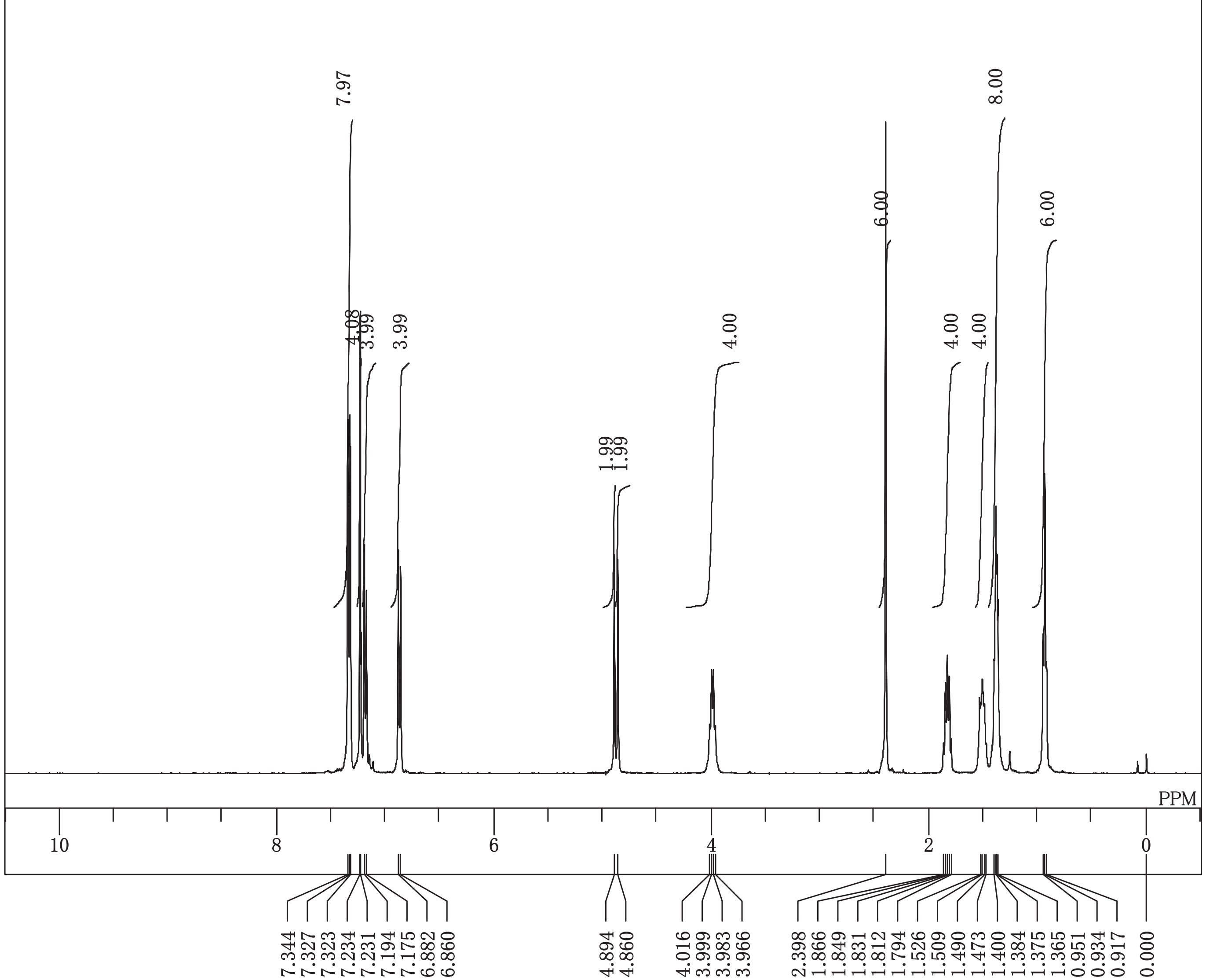

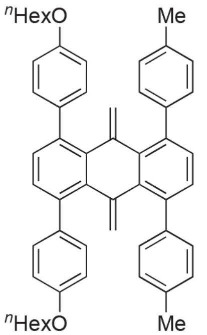

6ac

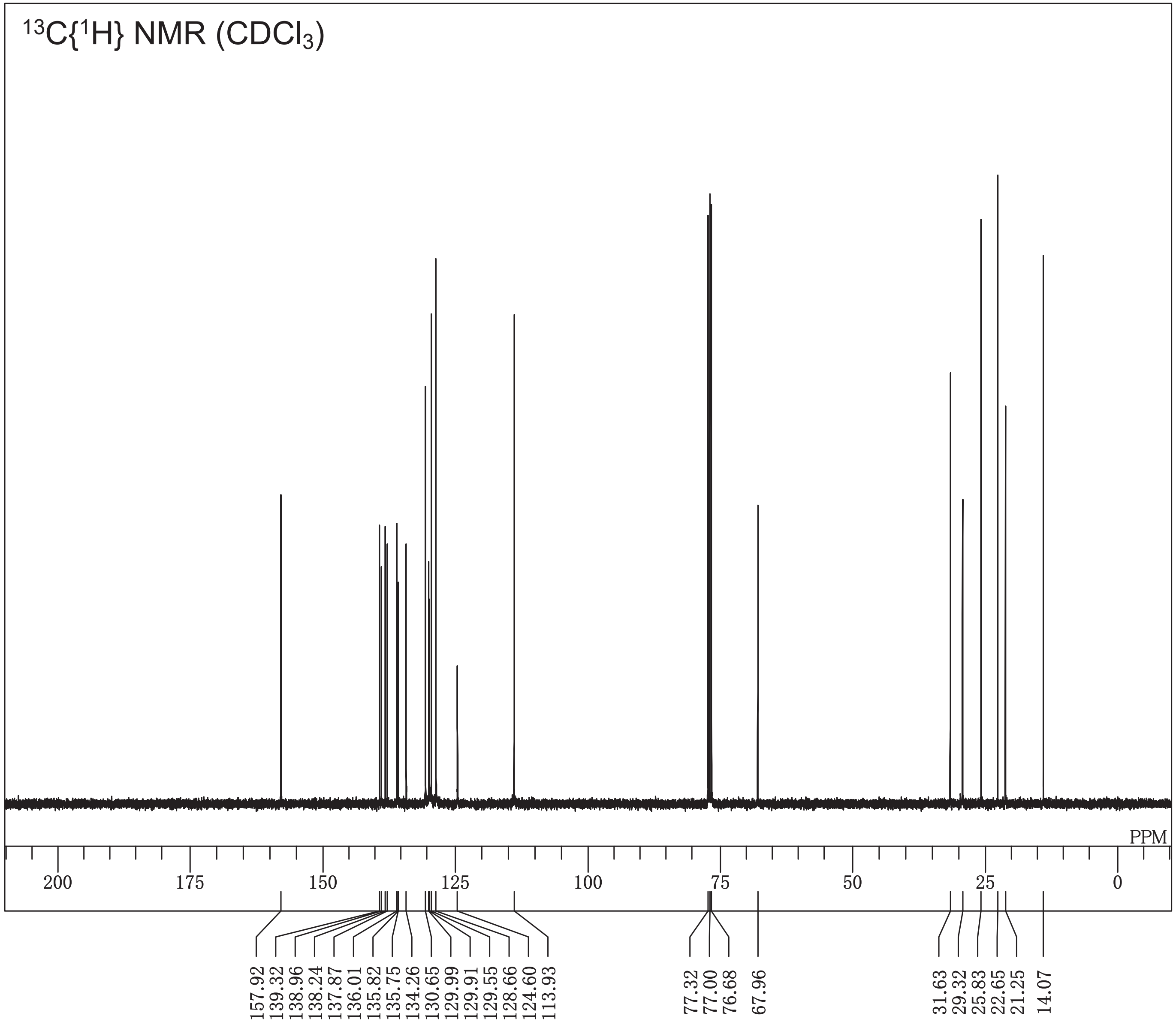

$^{13}C\{^1H\}$ NMR ($CDCl_3$)
PPM
200
175
150
125
100
75
50
25
0
157.92
139.32
138.96
138.24
137.87
136.01
135.82
135.75
134.26
130.65
129.99
129.91
129.55
128.66
124.60
113.93
77.32
77.00
76.68
67.96
31.63
29.32
25.83
22.65
21.25
14.07

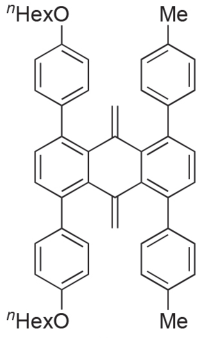

^{n}HexO
Me
^{n}HexO
Me

6ac

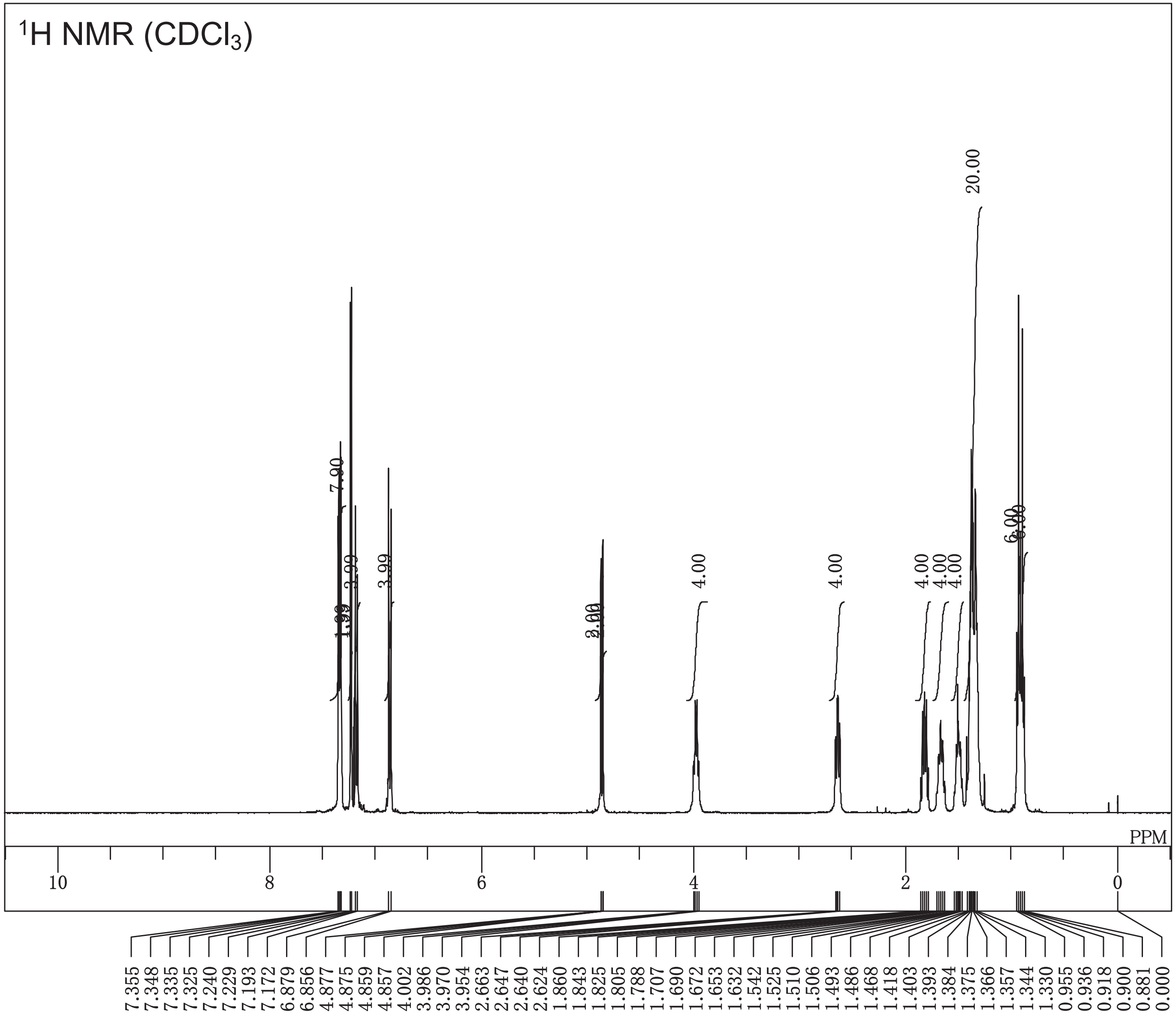

1H NMR (CDCl3)
7.90
1.99
2.99
3.99
2.00
4.00
4.00
4.00
4.00
4.00
20.00
6.00
6.00
PPM
10
8
6
4
2
0
7.355
7.348
7.335
7.325
7.240
7.229
7.193
7.172
6.879
6.856
4.877
4.875
4.859
4.857
4.002
3.986
3.970
3.954
2.663
2.647
2.640
2.624
1.860
1.843
1.825
1.805
1.788
1.707
1.690
1.672
1.653
1.632
1.542
1.525
1.510
1.506
1.493
1.486
1.468
1.418
1.403
1.393
1.384
1.375
1.366
1.357
1.344
1.330
0.955
0.936
0.918
0.900
0.881
0.000

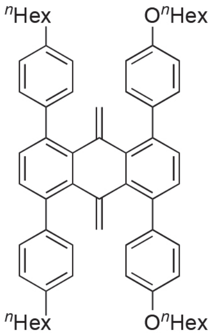

nHex
OnHex
nHex
OnHex

6ba

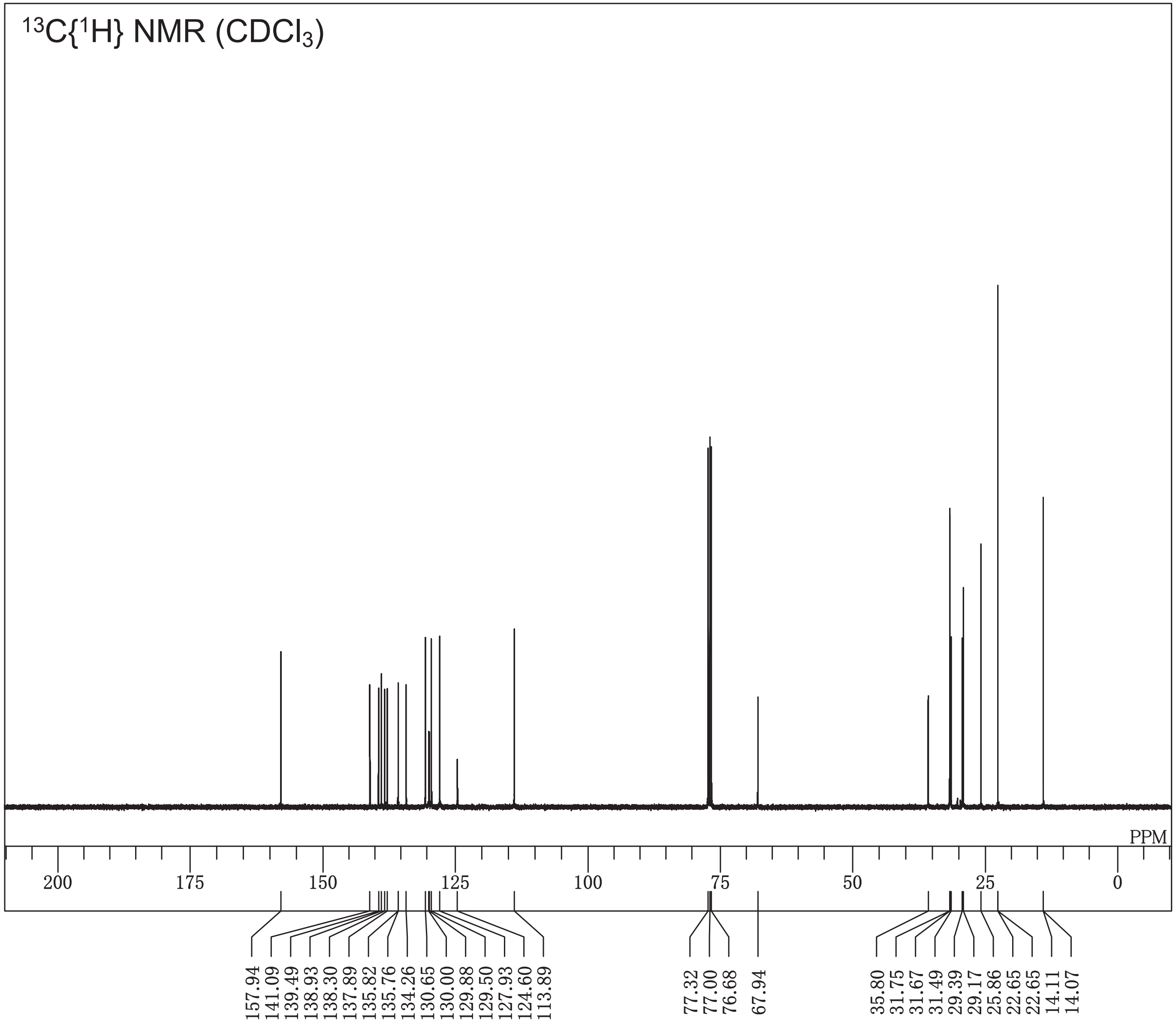
$^{13}C\{^1H\}$ NMR ($CDCl_3$)
PPM
200
175
150
125
100
75
50
25
0
157.94
141.09
139.49
138.93
138.30
137.89
135.82
135.76
134.26
130.65
130.00
129.88
129.50
127.93
124.60
113.89
77.32
77.00
76.68
67.94
35.80
31.75
31.67
31.49
29.39
29.17
25.86
22.65
22.65
14.11
14.07

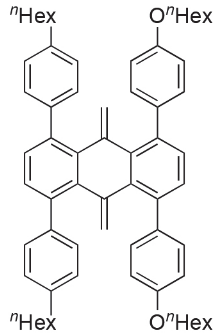
^{n}Hex
O^{n}Hex
^{n}Hex
O^{n}Hex

6ba

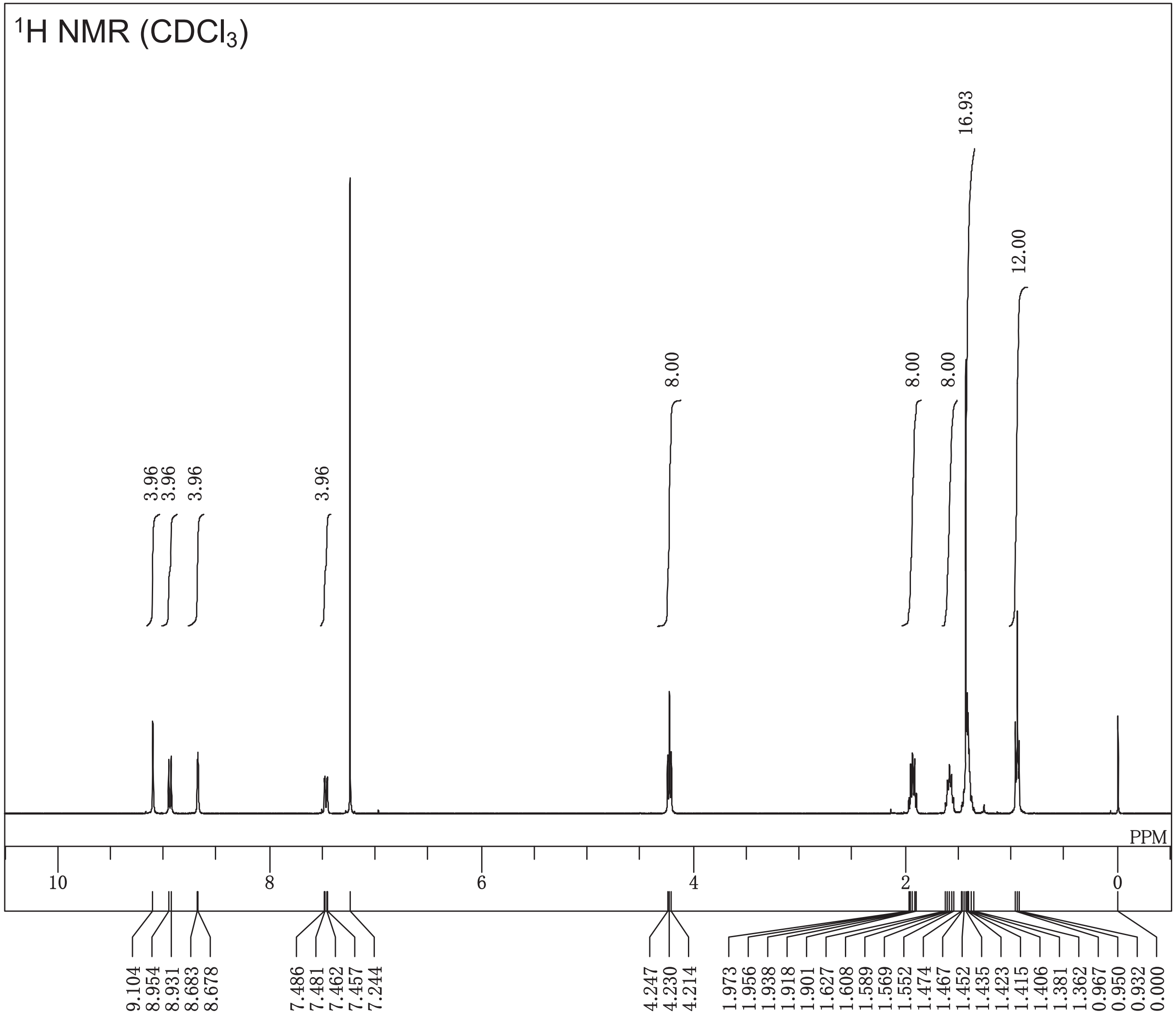

¹H NMR (CDCl3)
3.96
3.96
3.96
3.96
8.00
8.00
8.00
16.93
12.00
PPM
10
8
6
4
2
0
9.104
8.954
8.931
8.683
8.678
7.486
7.481
7.462
7.457
7.244
4.247
4.230
4.214
1.973
1.956
1.938
1.918
1.901
1.627
1.608
1.589
1.569
1.552
1.474
1.467
1.452
1.435
1.423
1.415
1.406
1.381
1.362
0.967
0.950
0.932
0.000

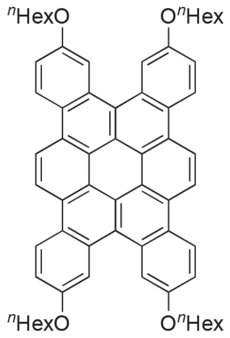

ⁿHexO
OⁿHex
ⁿHexO
OⁿHex

7aa

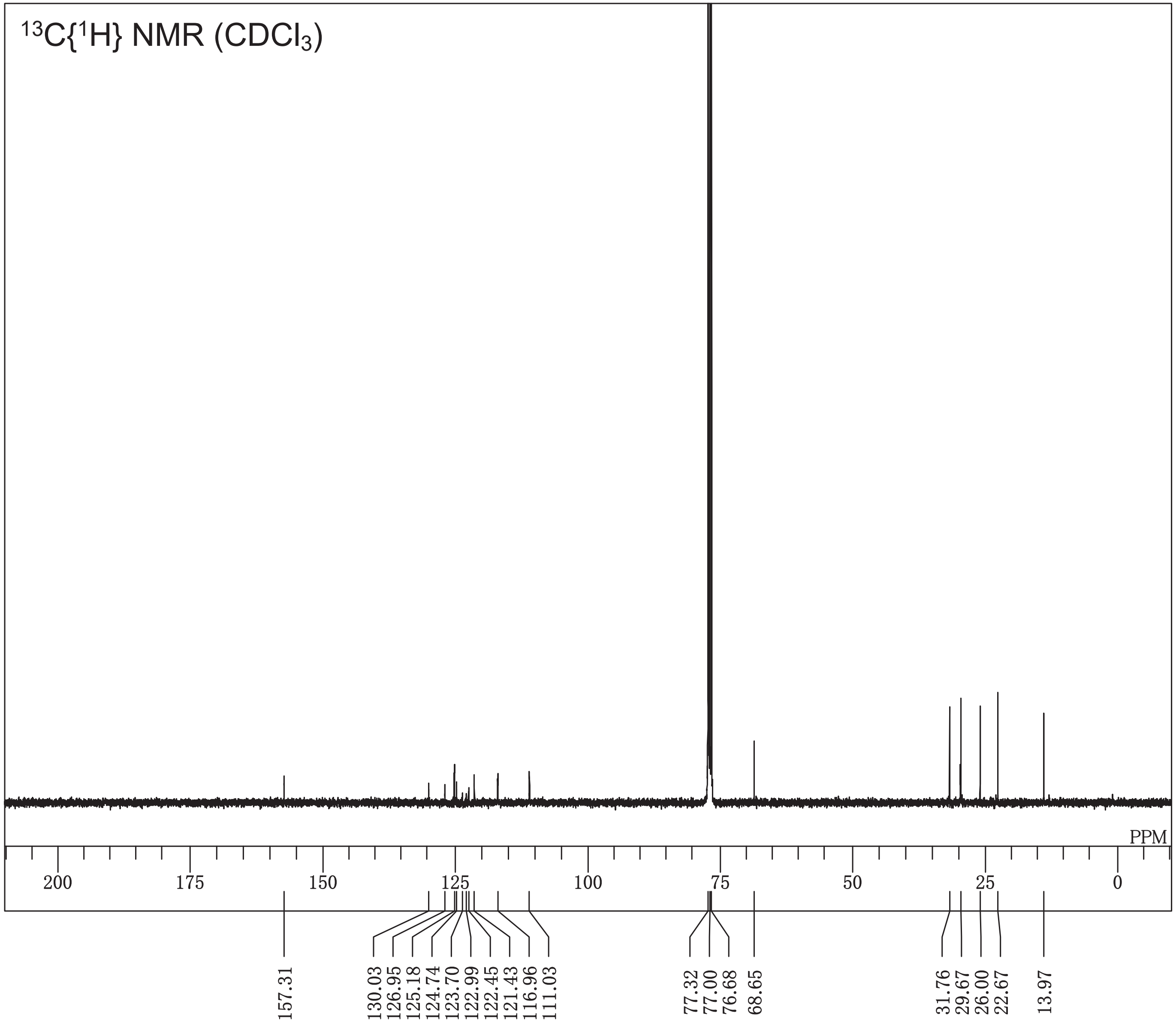

$^{13}C\{^1H\}$ NMR ($CDCl_3$)
PPM
200
175
150
125
100
75
50
25
0
157.31
130.03
126.95
125.18
124.74
123.70
122.99
122.45
121.43
116.96
111.03
77.32
77.00
76.68
68.65
31.76
29.67
26.00
22.67
13.97

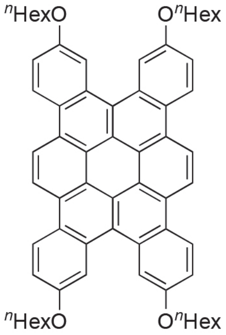

^{n}HexO
O^{n}Hex
^{n}HexO
O^{n}Hex

7aa

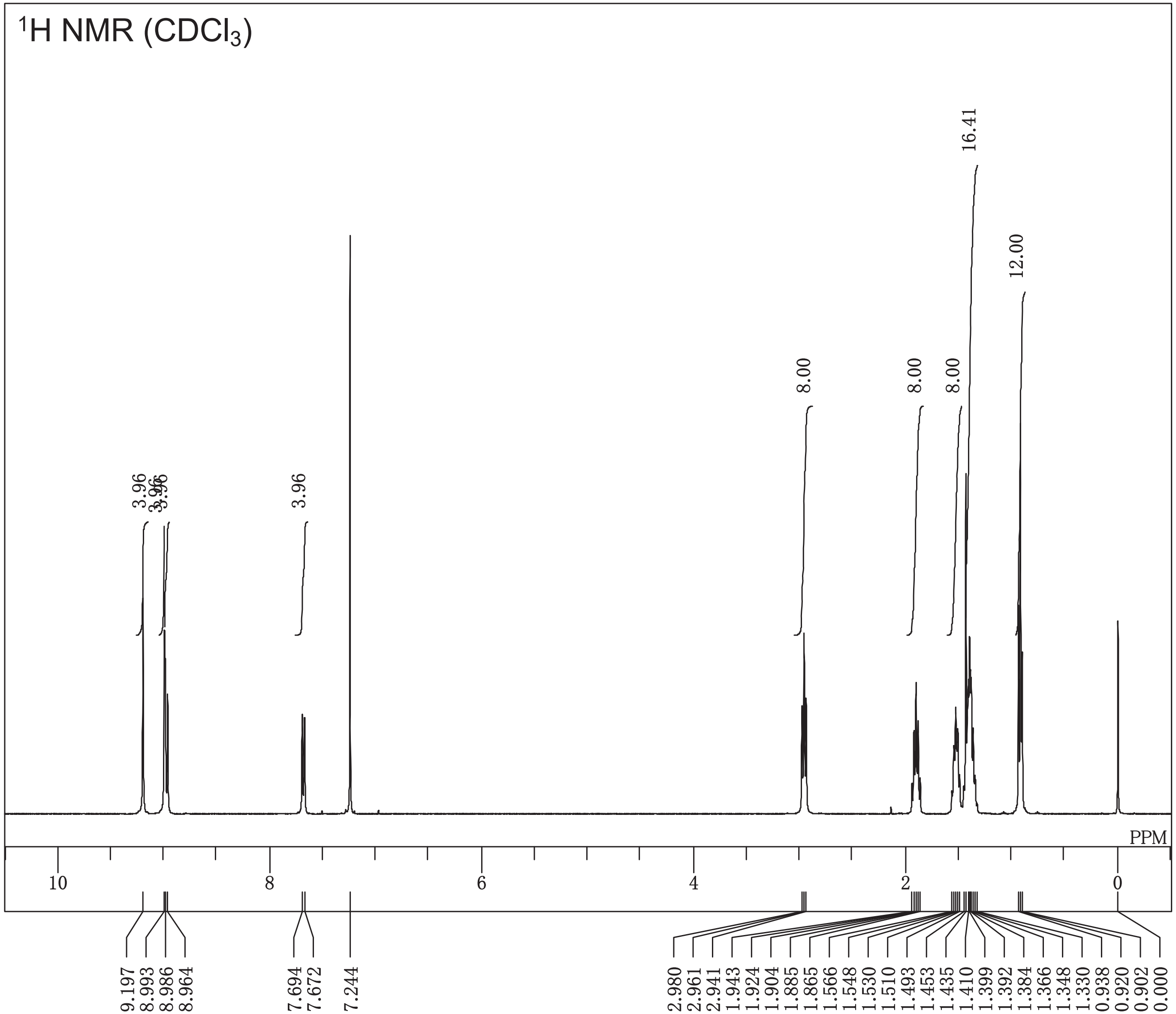
^1H NMR ($CDCl_3$)
3.96
3.96
3.96
3.96
8.00
8.00
8.00
16.41
12.00
PPM
10
8
6
4
2
0
9.197
8.993
8.986
8.964
7.694
7.672
7.244
2.980
2.961
2.941
1.943
1.924
1.904
1.885
1.865
1.566
1.548
1.530
1.510
1.493
1.453
1.435
1.410
1.399
1.392
1.384
1.366
1.348
1.330
0.938
0.920
0.902
0.000

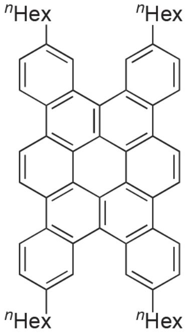
^{n}Hex
^{n}Hex
^{n}Hex
^{n}Hex

7bb

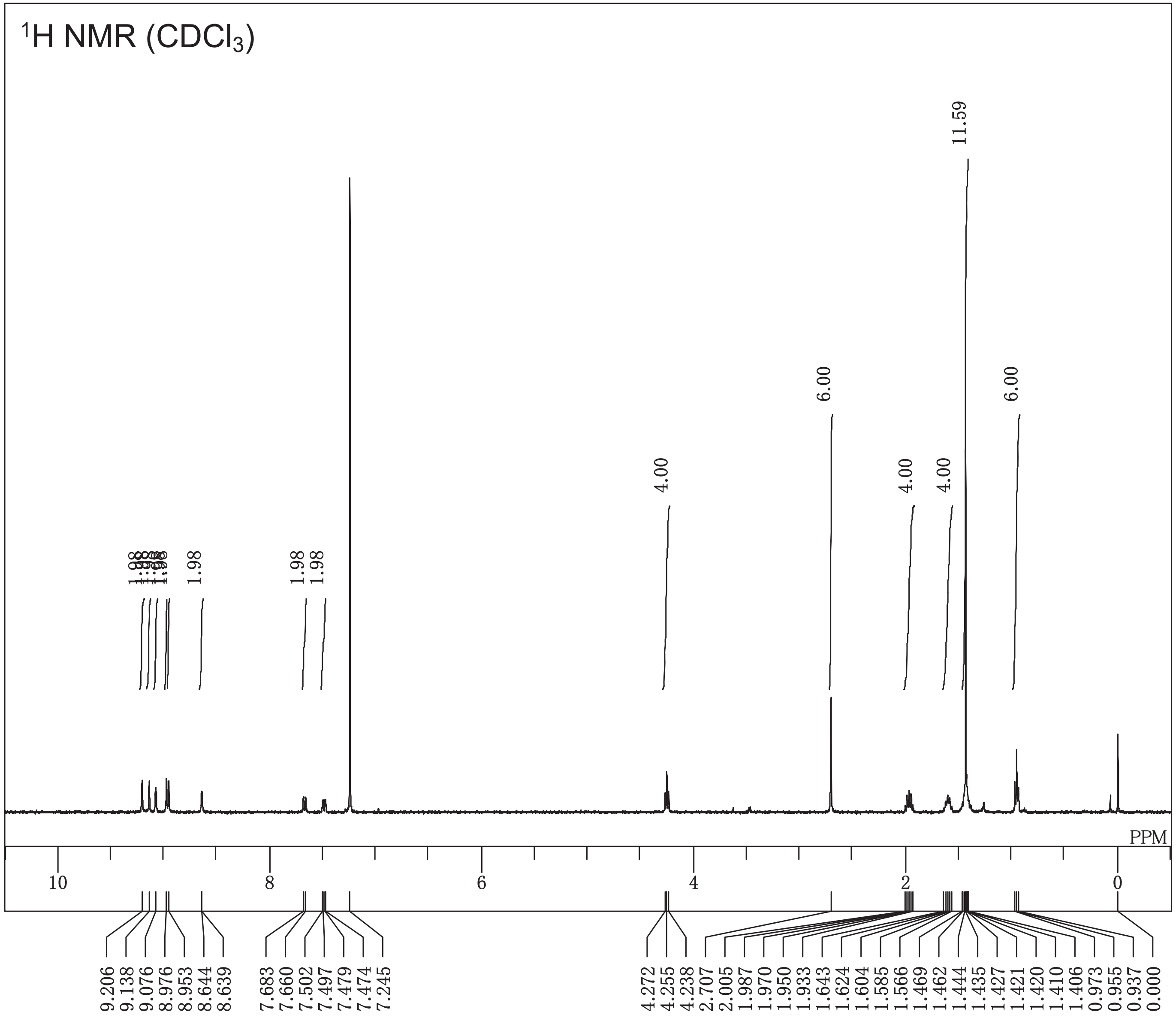

^{1}H NMR ($CDCl_3$)
1.98
1.98
1.98
1.98
1.98
4.00
6.00
4.00
4.00
11.59
6.00
PPM
10
8
6
4
2
0
9.206
9.138
9.076
8.976
8.953
8.644
8.639
7.683
7.660
7.502
7.497
7.479
7.474
7.245
4.272
4.255
4.238
2.707
2.005
1.987
1.970
1.950
1.933
1.643
1.624
1.604
1.585
1.566
1.469
1.462
1.444
1.435
1.427
1.421
1.420
1.410
1.406
0.973
0.955
0.937
0.000

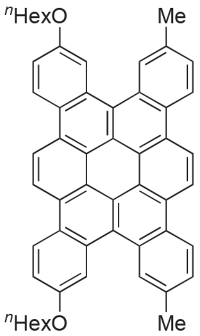

^{n}HexO
Me
^{n}HexO
Me

7ac

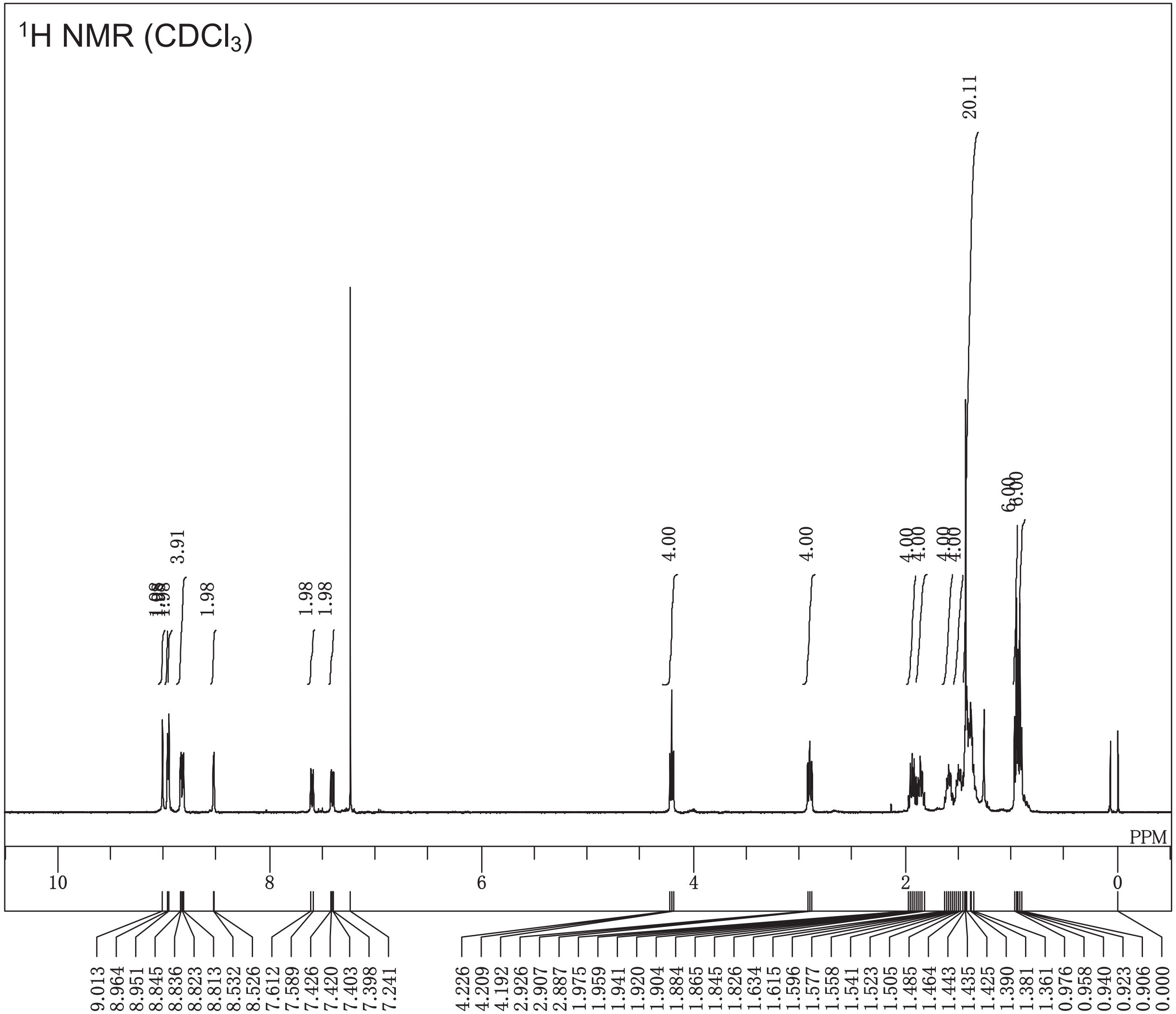

^{1}H NMR (CDCl$_3$)
20.11
6.00
6.00
4.00
4.00
4.00
4.00
4.00
4.00
1.98
1.98
1.98
3.91
1.98
1.98
PPM
10
8
6
4
2
0
9.013
8.964
8.951
8.845
8.836
8.823
8.813
8.532
8.526
7.612
7.589
7.426
7.420
7.403
7.398
7.241
4.226
4.209
4.192
2.926
2.907
2.887
1.975
1.959
1.941
1.920
1.904
1.884
1.865
1.845
1.826
1.634
1.615
1.596
1.577
1.558
1.541
1.523
1.505
1.485
1.464
1.443
1.435
1.425
1.390
1.381
1.361
0.976
0.958
0.940
0.923
0.906
0.000

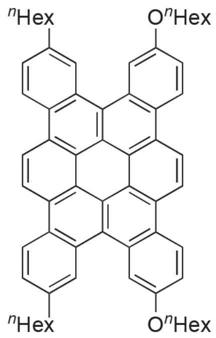

^{n}Hex
O^{n}Hex
^{n}Hex
O^{n}Hex

7ba

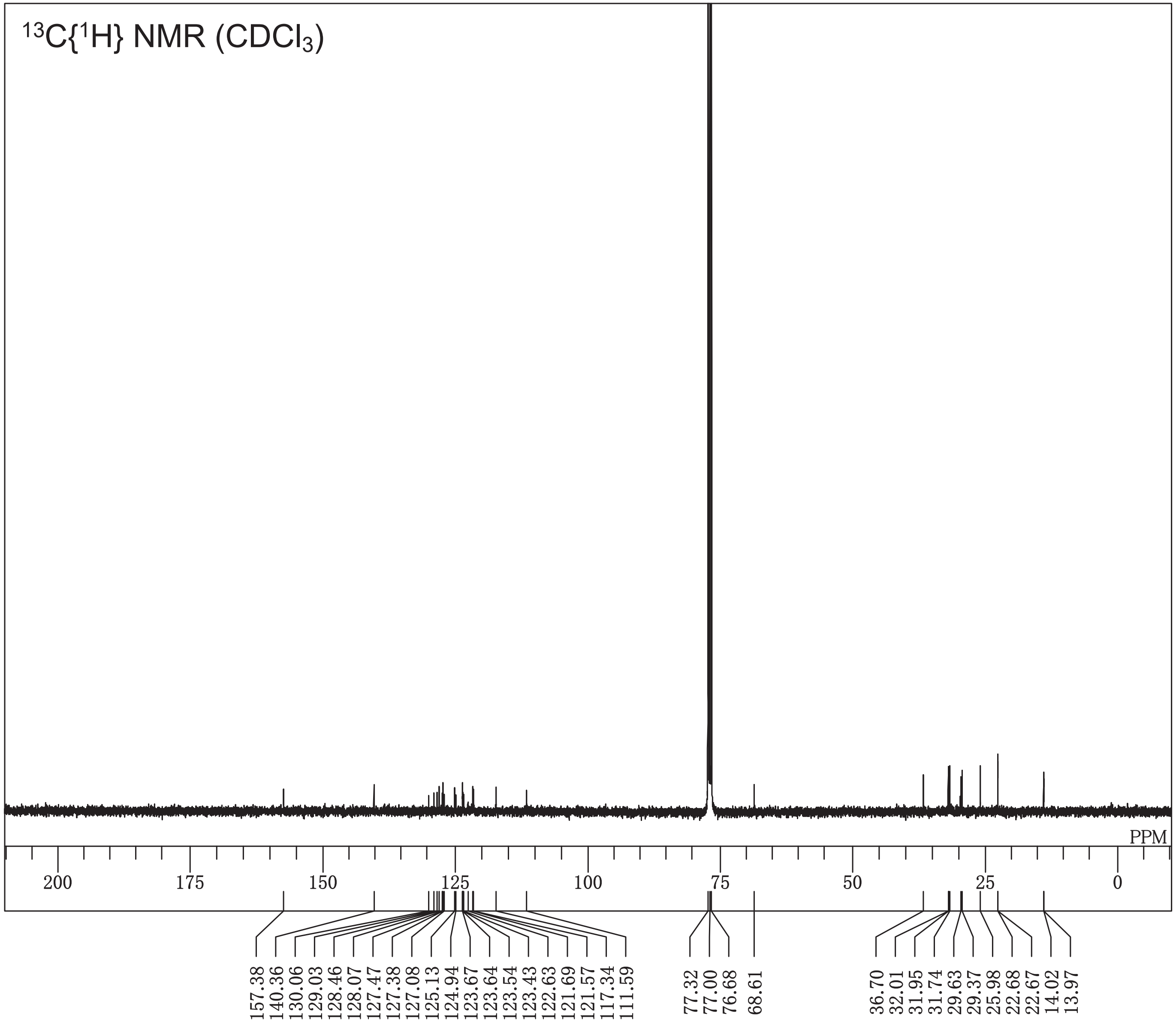

$^{13}C\{^1H\}$ NMR ($CDCl_3$)
PPM
200
175
150
125
100
75
50
25
0
157.38
140.36
130.06
129.03
128.46
128.07
127.47
127.38
127.08
125.13
124.94
123.67
123.64
123.54
123.43
122.63
121.69
121.57
117.34
111.59
77.32
77.00
76.68
68.61
36.70
32.01
31.95
31.74
29.63
29.37
25.98
22.68
22.67
14.02
13.97

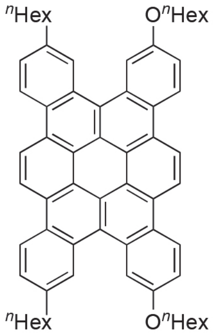

^{n}Hex
O^{n}Hex
^{n}Hex
O^{n}Hex

7ba
